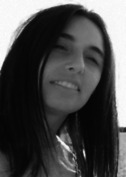# Green Chemistry Meets Asymmetric Organocatalysis: A Critical Overview on Catalysts Synthesis

**DOI:** 10.1002/cssc.202100573

**Published:** 2021-06-23

**Authors:** Achille Antenucci, Stefano Dughera, Polyssena Renzi

**Affiliations:** ^1^ Department of Chemistry University of Turin Via Pietro Giuria, 7 10125 Turin Italy; ^2^ NIS Interdeprtmental Centre INSTM Reference Centre University of Turin Via Gioacchino Quarello 15/A 10135 Turin Italy

**Keywords:** catalyst synthesis, *E* factor, green chemistry, organocatalysis, sustainability

## Abstract

Can green chemistry be the right reading key to let organocatalyst design take a step forward towards sustainable catalysis? What if the intriguing chemistry promoted by more engineered organocatalysts was carried on by using renewable and naturally occurring molecular scaffolds, or at least synthetic catalysts more respectful towards the principles of green chemistry? Within the frame of these questions, this Review will tackle the most commonly occurring organic chiral catalysts from the perspective of their synthesis rather than their employment in chemical methodologies or processes. A classification of the catalyst scaffolds based on their *E* factor will be provided, and the global *E* factor (*E*_G_ factor) will be proposed as a new green chemistry metric to consider, also, the synthetic route to the catalyst within a given organocatalytic process.

## Graphical abstract

***E*****factor under scrutiny**: Do you know which is the impact on the overall process of the organocatalyst synthesis? Within the framework of the green chemistry principles, this Review analyses the synthetic routes towards some of the most important organocatalyst scaffolds. The introduction of a new chemistry metric, the *E*_G_ factor, will provide an idea of the actual impact of the catalyst synthesis within the overall organocatalytic process.

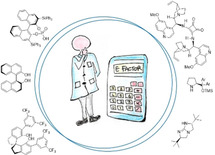

## 1 Introduction

**Figure 1:**
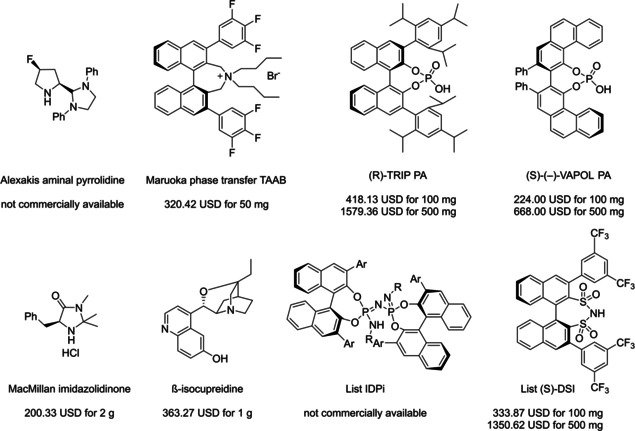
Some complex molecular scaffolds employed in organocatalysis.^[10]^

Among the three major branches of asymmetric catalysis, namely bio‐, organometallic and organo‐catalysis, the latest has exponentially gained attention and importance just starting from the end of 1999.^[1]^ Despite the presence of isolated examples of asymmetric catalysis from small organic molecules in the early literature,^[2]^ the well‐defined area of asymmetric organocatalysis has emerged thanks to the elucidation of general activation modes and to its broad applicability. Apart from the novelty, several key factors have given a significant contribution to the widespread success of chiral organic scaffolds as catalysts, such as: biodegradability, general insensitivity towards oxygen and moisture, availability from natural sources and generally lower cost with respect to enzymes or transition metals. All of these key factors give a nod to the twelve principles of green chemistry,^[3]^ and it is worth noticing that both green chemistry and asymmetric organocatalysis were emerging and relatively unexplored research areas, growing side by side more or less simultaneously. Anyway, a mutual interconnection between the two areas, from a formal point of view, is still missing. While the constant demand for more eco‐sustainable protocols still makes green chemistry an attractive research field, nowadays asymmetric organocatalysis seems to have fully exploited its potential. In fact, if compared to the mole of catalysts and protocols provided by academia, industrial applications are relatively scarce.^[4]^ This is not surprising if the cost and the synthetic efforts to prepare some classes of organocatalysts are taken into account (see Figure 1 for complex molecular scaffolds),^[5]^ making inconvenient their employment on a large scale.

If in the original definition an organocatalyst is a small organic molecule having no metal as part of the “active principle”,^[6]^ no limitation is imposed to the employment of transition metals, toxic reagents, or difficult reaction conditions in the synthetic route towards the target organic compound. Moreover, in the last years to answer to the specific request for high reactive and enantioselective compounds, organocatalysis has moved more and more towards the development of catalysts with very complex structures without taking inspiration from sustainability. Curiously, the three pioneering papers of modern asymmetric organocatalysis present three complementary aspects with reference to the complexity of the catalyst scaffold.^[7]^ Thus a discrepancy between organocatalysis and green chemistry may arise.

Within this background, our aim will be to critically analyze the synthetic routes followed to prepare organocatalyst scaffolds, providing the reader with the tools to evaluate these synthetic processes based on the twelve principles of green chemistry.^[3]^ A classification of the catalyst scaffolds based on their *E* factor will be provided in order to give a trusted general idea of the impact of the synthetic process towards the catalyst.^[8]^ Indeed, the *E* factor appears to be the most complete mass‐based parameter to evaluate the overall greenness in the context of an entire process, also in the perspective of industrial applications. On the contrary, other mass‐based metrics would neglect other important waste sources (e. g., reaction mass efficiency) or would lead to situations of ambiguity in the definition of non‐benign reagents (e. g., effective mass yield). On the contrary, impact‐based metrics like the EcoScale would likely lead to the calculation of meaningless negative scores. In fact, it must be stressed that most of the synthetic routes to organocatalyst scaffolds consist in multiple steps and have not been designed in the perspective of sustainability. As highlighted in Chapter 8, a global *E* factor (*E*_G_ factor) will be proposed as a new green chemistry metric to consider, also, the synthetic route towards the catalyst within a given organocatalytic process. Organocatalyst scaffolds will be divided according to the general classification by List and Maruoka in: Lewis bases, Brønsted bases, Brønsted acids and Lewis acids.^[6]^

Additional insights will be dedicated, in separate chapters, to hydrogen bonds and phase‐transfer organocatalysts. Green chemistry related to the process analyzed will be discussed. Moreover, solvents employed will be evaluated according to the solvent sustainability guide, while hazard classification from European Union and ECHA will be reported for the most used reagents.^[9]^ In any case, it must be stressed that this critical review does not intend to diminish the importance of organocatalysts with very complex structures. In our opinion a major inspiration of catalyst design to the twelve principles of green chemistry in the future may help to develop really sustainable processes.

## 2 Lewis Base Catalysts

### 2.1 Prolines, imidazolidinones, and other amino acid derivatives

The first chapter of this review is dedicated to those catalysts which, according to List and Maruoka, can be classified as Lewis bases. Between them, proline and derivatives boosted a rapid and extraordinary development of this field, making aminocatalysis the privileged method for the asymmetric functionalization of carbonyl compounds.^[11]^

**Figure 2:**
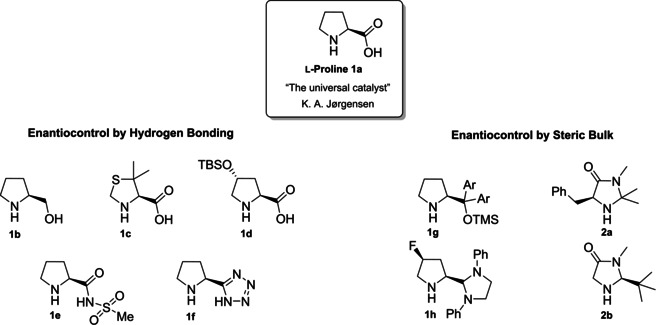
Classification of catalysts derived from 1 a.

From the infancy of aminocatalysis, the natural amino acid proline (**1 a**) has assumed a central role as to be considered both as the “simplest enzyme” in nature^[12]^ and as “the universal catalyst”.^[13]^ In fact, because of its versatility, efficiency and generality, **1 a** has been extensively applied as the privileged catalyst in the nucleophilic addition or substitution of carbonyl compounds, imines, azodicarboxylates and nitrosobenzenes with a variety of electrophiles.[^11a^, ^11b^, ^11c^, ^14^] Moreover, being a natural and renewable compound, **1 a** fully meets the principles of green chemistry.^[15]^ Considering the catalytic power of natural product **1 a**, chemists dedicated a lot of efforts to the development of its derivatives to improve the solubility, enhance the acidity or increase the steric hindrance of the directing group (Figure 2). The stereoinduction in the product can be controlled through hydrogen‐bond interactions or steric hindrance,^[16]^ thus catalysts derived from **1 a** can be divided into two main groups.^[17]^ The first group is characterized by a hydrogen‐bond donor substituent, able to activate and position the electrophile, while the catalysts from the second group present a bulky moiety allowing the electrophile to approach the enamine only from one side (Figure 2).

**Scheme 1:**
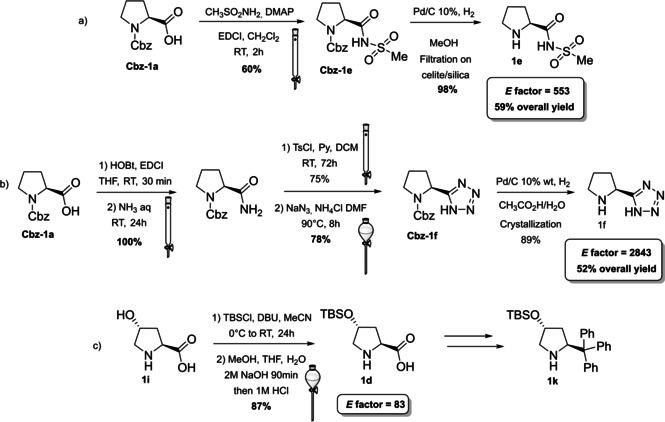
Synthesis of selected proline derivatives bearing hydrogen‐bond‐directing groups (DMAP= 4‐dimethylaminopyridine, DCM= dichloromethane, EDCl= 1‐(3‐dimethylaminopropyl)‐3‐ethylcarbodiimide, DBU= 1,8‐diazabicyclo[5.4.0]undec‐7‐ene, TBSO= *tert*‐butyldimethylsilyl ether).

Compounds **1 b**–**1 f** preserve the central proline moiety, in which a tunable hydrogen‐bond donor is introduced to increase the solubility and enhance the acidity. In general, these catalysts revealed to be more reactive and more enantioselective in specific reactions, but the generality of **1 a** was lost. Between all the structures developed, we decided to present the synthesis of the three derivatives depicted in Scheme 1. In accordance with the 2^nd^ principle, the incorporation of the starting material is maximized, in fact catalysts **1 d**, **1 e**–**1 f** are obtained through lateral derivatization of a natural compound, thus preserving its original stereochemistry.

The groups of Yamamoto and Ley, independently, contributed to this field introducing proline derivatives bearing a tetrazole and an acyl sulfonamide group (Scheme 1a, b, catalysts **1 e** and **1 f**).[^18^, ^19^] The tetrazole and the sulfonamide moieties are employed to replace proline carboxylic acid function in order to obtain a more soluble catalyst with preserved (**1 f**) or increased acidity (**1 e**). In both cases, protection of the secondary amine functionality with *N*‐carboxybenzyl group (Cbz) is applied to direct the reaction towards the carboxylic group of **1 a**. The 8^th^ principle of green chemistry is not taken into account, since protection/deprotection strategies were not minimized or excluded. The synthesis of compound **1 e** is quite straightforward, and it is based on the coupling of protected‐proline Cbz‐**1 a**, in classic reaction conditions, with a sulfonamide (Scheme 1a). The Cbz removal is then realized through hydrogenolysis. Product **1 e** is obtained in around 60 % yield and one column chromatography can be avoided employing crude Cbz‐**1 e** in the hydrogenation step. In order to remove palladium on carbon (Pd/C), the sulfonamide **1 e** should be filtered through a short pad of silica and celite. An *E* factor of 533 is obtained. The introduction of the tetrazole moiety is lengthier and requires five synthetic steps, two column chromatographies, one extractive workup and one crystallization, affording catalyst **1 f** in 52 % yield with an *E* factor of 2843 (Scheme 1b). The carboxylic group of Cbz‐proline is first converted in an amide and then to a cyano group, which allows the introduction of the tetrazole after reaction with sodium azide. Finally, Cbz protecting group is removed.^[18]^ Both synthetic procedures go against the 3^rd^, 4^th^, and 5^th^ principles of green chemistry employing volatile organic compounds (VOC) solvents and reagents, which are flammable (hydroxybenzotriazole (HOBt), DMF), from harmful to very toxic for aquatic life (HOBt, NaN_3_, Pd, NH_3_) and that can cause damage to fertility and to unborn child (DMF).

**Figure 3:**
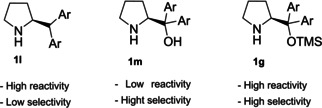
Comparison between methylpyrrolidine 1 l, diarylprolinol 1 m, and diarylprolinol ether 1 g.

The last derivative of proline, that we present, is obtained from *trans*‐4‐hydroxy‐*L*‐proline (**1 i**), a natural non‐conventional amino acid that can be isolated by hydrolyzed gelatin in accordance with the 7^th^ principle. The same **1 i** can be used as catalyst after a simple protection of the hydroxyl group as silyl ether (Scheme 1c). The protected *trans*‐4‐hydroxy‐L‐proline derivative **1 d** is obtained in 87 % yield after a two‐step reaction, one extractive workup, and precipitation, resulting in a low *E* factor of 83. Compound **1 d** can be further derivatized and converted to catalyst **1 k** belonging to the second group (steric bulk control).^[20]^ Between catalysts that can exert enantiocontrol through steric bulk, diarylprolinol ethers and imidazolidones are the most exploited.^[21]^ For this reason, we will analyze their synthesis and evolution. Jørgensen and Hayashi groups, independently, contributed to the development of diarylprolinol ethers **1 g**.^[22]^ These compounds originated from the need to find a catalyst combining both high reactivity and high selectivity (Figure 3). As the authors told, the inspiration came from two related compounds: methylpyrrolidine (**1 l**) and diarylprolinol (**1 m**) both bearing proline skeleton. Compound **1 l** is characterized by high reactivity but low selectivity probably due to an insufficient steric shielding; on the contrary **1 m** is highly selective but poorly reactive. The simple protection of the OH group as silyl ether prevents oxazolide formation delivering a catalyst, which is at the same time highly reactive and enantioselective.

**Scheme 2:**
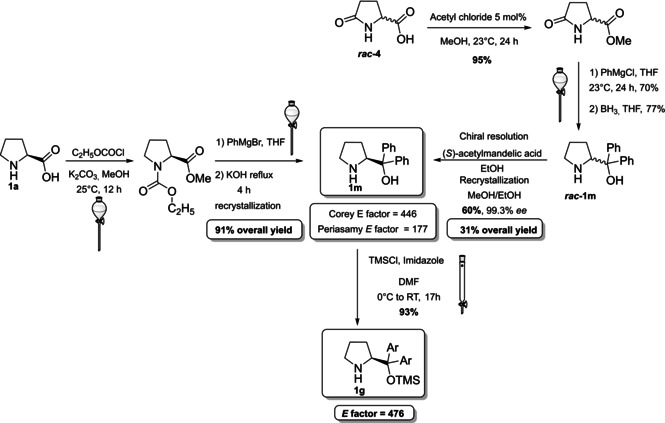
Synthesis of selected proline derivatives bearing sterically hindered directed groups.

Different synthetic routes have been proposed to access **1 m**, from which the most active diarylprolinol ether (**1 g**) can be prepared in one step (Scheme 2). As summarized in Scheme 2, **1 m** can be obtained from two different multi‐step approaches employing the natural compounds **1 a** or racemic pyroglutamic acid (**4**) as starting materials. Bhaskar Kanth and Periasamy proposed a three‐step synthesis starting from **1 a** in which the amine functionality is protected as carbamate with ethyl chloroformate; at the same time, the carboxylic group is esterified with methanol. In the next step, a Grignard reagent is exploited to introduce the diphenyl functionality on the carboxylic group. Deprotection and crystallization delivers product **1 m** in 91 % yield. A low *E* factor of 177 can be calculated from this process considering that all the intermediates are purified by extractive workup avoiding production of waste due to chromatography.^[23]^

Nevertheless, the usage of ethyl chloroformate and phenylmagnesium bromide may cause risks and concerns for large scale production, considering that these compounds are highly flammable, they can cause skin burns and eye damage. Moreover, ethyl chloroformate is fatal if inhaled and phenylmagnesium bromide may cause genetic defects and cancer (data from ECHA).^[24]^

The synthetic path developed by Corey et al. employs as starting material racemic pyroglutamic acid (***rac***‐**4**), a natural amino acid derivative. The synthesis of diarylprolinol (**(*S*)‐1 m**) is accomplished by a three‐step sequence (esterification, reaction with phenylmagnesium chloride, and reduction with borane) followed by resolution of ***rac***‐**1 m** with (*S*)‐(+)‐*O*‐acetylmandelic acid and recrystallization (Scheme 2). Access to the catalyst enantiomer **(*R*)‐1 m** is possible employing as resolution reagent (*R*)‐(−)‐*O*‐acetylmandelic acid.^[25]^ All reactions are run at room temperature observing the 6^th^ principle and, despite the employment of a resolution reagent does not meet the 9^th^ principle, the chosen one is a natural compound derivative. As concern the safety, acetyl chloride is less toxic than ethyl chloroformate but still highly flammable and borane cannot be considered a green reagent, being also harmful for aquatic life. This process is characterized by an *E* factor of 466, higher than the one obtained by Bhaskar Kanth and Periasamy,^[23]^ but if one takes into account the cost of the process, Corey path is probably more appealing considering that both racemic and *L‐*pyroglutamic acid are less expensive than **1 a** (100 g of racemic pyroglutamic acid **4** costs 66.84 USD; 100 g of *L*‐pyroglutamic acid costs 73.92 USD; 100 g of **1 a** costs 208.94 USD from Sigma–Aldrich/Merck). For what concerns **1 g**, its synthesis can be easily realized incorporating the TMS group in a single step starting from the corresponding prolinol **1 m** (Scheme 2).^[22a]^ Because of the need for a repeated extractive workup and a chromatographic purification the *E* factor rises to 476, starting from **1 a**.

**Scheme 3:**
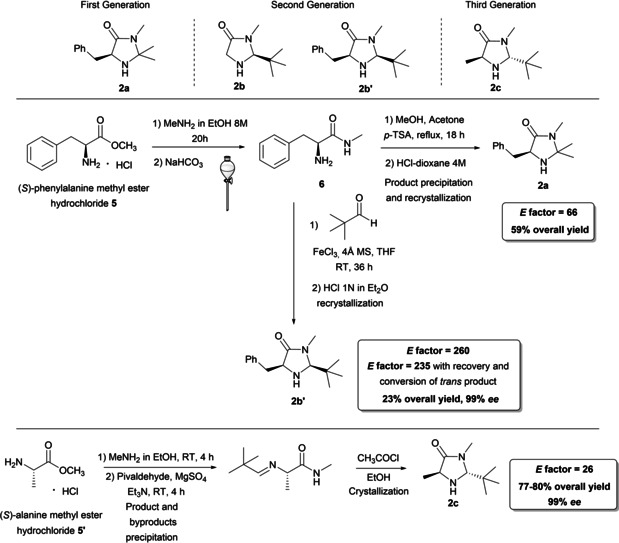
Synthesis of MacMillan imidazolidinones belonging to the 1^st^, 2^nd^ and 3^rd^ generation.

Together with enamine catalysis, iminium ion activation allows the asymmetric functionalization of a variety of unsaturated carbonyl compounds. In this case the primary role of catalyst is occupied by MacMillan's imidazolidinones **2**, which are able to form a reversible and reactive iminium ion, ensuring stereocontrol from π‐facial discrimination. MacMillan's imidazolidinones **2** can be classified as 1^st^, 2^nd^ and 3^rd^ generation catalysts (Scheme 3), differing from the presence of a hydrogen or substituents in the α‐positions to the secondary amine group.[^7b^, ^26b^, ^26c^] Second‐generation compounds have been introduced by MacMillan and co‐workers to increase imidazolidinone reactivity in the presence of furan and indole substrates. In fact, the removal of one of the methyl group from the 1^st^ generation compounds allows the catalyst to form the iminium ion more straightforwardly. At the same time, the substitution of the other methyl with a more hindered *t*‐butyl group increases the coverage of the blocked *Si*‐enantioface, thus obtaining stereocontrol. 1^st^ and 2^nd^ Generation imidazolidinone, according to MacMillan protocol, can be obtained from a common intermediate which is synthesized from (*S*)‐phenylalanine methyl ester **5**, an amino acid derivative, through amide bond formation (Scheme 3). The amide **6** can be cyclized in the presence of acetone and an acidic catalyst to give imidazolidinone **2 a** (first generation) in 59 % yield after acidification. The simple workup consisting of extraction, product precipitation, and recrystallization contributes to a very low *E* factor of 66.

On the contrary, for the 2^nd^‐generation compounds e. g. **2 b’**, the *E* factor rises to 260. The approach pursued is the same, but the lower overall yield (23 % versus 59 %) contributes to the *E* factor increase. The value can be reduced to 235 if the *trans* product is recovered and converted to *cis*‐imidazolidinone (**2 b’**) through elimination reaction in the presence of NaHCO_3_ and FeCl_3_, in THF as the solvent. Attention should be paid in the employment of methylamine (can cause serious eye and skin damage and respiratory irritation, contains gas under pressure and may explode if heated) and FeCl_3_ which may be corrosive to metals, causes skin irritation and may cause an allergic skin reaction although being a fully abundant natural compound. Few and medium issue solvents (MeOH, EtOH, acetone) are applied in two out of four synthetic steps to 1^st^ generation imidazolidinones. A step forward enamine catalysis and the merge between organo‐ and photoredox catalysis have been made possible through the development of 3^rd^ generation imidazolidinones.^[27]^ These compounds are characterized by a reduced steric hindrance because of the *trans* arrangement of substituents. Despite the similar synthetic approach, **2 c** is obtained from alanine methyl ester hydrochloride in higher yield with respect to compounds **2 a** and **2 b**. The lack of intermediates purifications and chromatography results in a very low *E* factor of 26.^[28]^ Recent developments towards recoverable MacMillan catalysts **2** have been summarized by Deepa and Singh.^[29]^

**Scheme 4:**
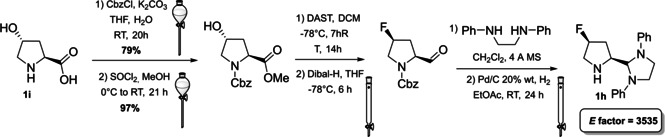
Synthesis of modular 1 h (DAST= diethylaminosulfur trifluoride, Dibal‐H= diisobutylaluminium hydride).

The last example we present is the modular aminal–pyrrolidine (**1 h**) designed by the group of Alexakis (Scheme 4).^[30]^ This organocatalyst is characterized by a pyrrolidine moiety in which an aminal group is introduced to increase steric bulk. The aminal moiety can, in fact, accommodate different substituents making this structure modular. Moreover, the introduction of a group at the 4‐position of the pyrrolidine moiety, like a fluorine atom, is able to stabilize one catalyst conformation, allowing efficiency and enantioselectivity improvement. Alexakis and co‐workers proposed a multi‐step synthesis starting from natural product *trans*‐4‐hydroxy‐*L*‐proline (**1 i**) (Scheme 4). In the first two steps, the amine functionality is protected with CbzCl and the carboxylic acid is esterified with methanol. Then, a nucleophilic substitution at low temperature is exploited to introduce the fluorine atom and invert the configuration of the stereogenic center. The ester is reduced to aldehyde at −70 °C, allowing the introduction of aminal group after reaction with a diamine. According to the diamine employed, it is possible to modulate the catalyst structure. The application of two cryogenic steps is not in line with the 6^th^ principle of green chemistry. In the last step the Cbz protecting group is removed and product **1 h** is obtained in 61 % overall yield. Considering the presence of two column chromatography purifications and the absence of sequential reactions in one solvent, the *E* factor rises to 3535.

**Scheme 5:**
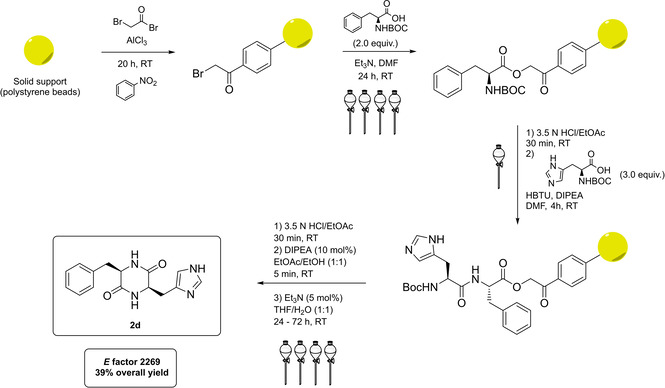
Synthesis of 2 d (HBTU= 3‐[Bis(dimethylamino)methyliumyl]‐3H‐benzotriazol‐1‐oxide hexafluorophosphate, DIPEA= *N*,*N*‐diisopropylethylamine).

Although organocatalysis is strictly referred to small organic molecules, synthetic peptides represent a very interesting class of compounds for asymmetric synthesis, especially because of their easiness of preparation and availability of starting materials.^[31]^ Between them, α,α‐ and α,β‐dipeptides are those finding a practical synthetic application. Among the very first examples of dipeptide organocatalysts,^[32]^ Inoue and co‐workers proposed an interesting *cyclo*‐*L*‐phenylalanine‐*L*‐histidine (**2 d**) for the asymmetric Strecker reaction. A recent approach to **2 d** includes polystyrene‐supported Merrifield‐type solid‐phase synthesis, followed by deprotection from support and cyclization in mild reaction conditions,^[33]^ as depicted in Scheme 5.

Throughout the synthesis of this catalyst, it is remarkable the lack of any chromatographic purification, an interesting point in the aim of reducing the total waste amount. Unfortunately, the need for several extractions and washing steps with classical VOCs contributes to a high final *E* factor value of 2269. Nevertheless, as a starting point for a green improvement of the procedure some important key features can be identified, such as the use of naturally occurring amino acids as the substrates and the recyclability of the solid support. Moreover, the volatile solvents employed are among the less environmentally hazardous (e. g., EtOAc, EtOH) or possess suitable green alternatives (e. g., 2‐MeTHF instead of CH_2_Cl_2_ or THF). Very recently, even DMF was shown to be dispensable for solid phase peptide synthesis, in favor of *N*‐octyl pyrrolidone.^[34]^

**Scheme 6:**
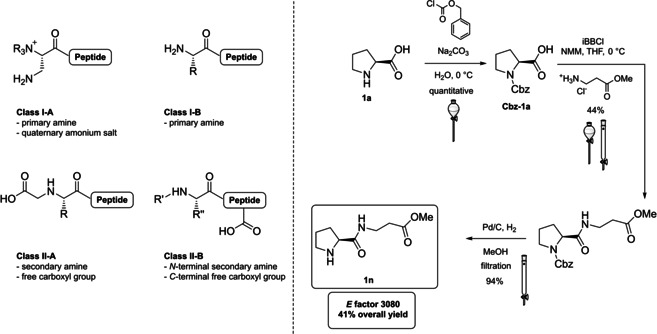
Design of potential peptide catalysts and synthesis of Juaristi catalyst 1n for the neat aldol reaction under ball milling conditions (iBBCl*= iso*butyl chloroformate, NMM= 4‐methylmorpholine).

For what concerns classical dipeptides, the lowest molecular weight subclass of polypeptide catalysts, their design has been inspired by Type I aldolases. According to Reymond and co‐workers,^[35]^ peptides catalysts can be classified in four main classes and divided in class I, if based on terminal primary amines, and class II peptides if based on secondary amines. A quaternary ammonium salt or a free carboxyl group can be present as displaced in Scheme 6.

Being inspired by Type I aldolases, it is not surprising that the aldol reaction was the first transformation on which peptide organocatalysts were tested. With the development of this branch of catalysis, several different peptides turned out to be excellent chiral organocatalysts,^[36]^ including green approaches.^[37]^ However, proline‐based α,α‐ and α,β‐dipeptides undoubtedly preserved their privileged role.^[38]^ Herein, we describe the route towards catalyst **1 n** by Juaristi and co‐workers, an α,β‐proline‐based dipeptide applied to the neat aldol reaction under ball milling conditions (Scheme 6).^[37d]^ Despite the design of catalyst **1 n** has not been explicitly inspired by the twelve principles, naturally occurring *L*‐proline and *L*‐alanine are employed as the starting materials (7^th^ principle), warming or extreme cooling are avoided (6^th^ principle) and catalyst **1 n** is employed to promote and accelerate heterogeneous reactions (9^th^ principle). Room for improvement is offered by the possibility of replacing reaction solvents (namely, THF and methanol) with their greener counterparts 2‐MeTHF and ethanol, respectively. Unfortunately, two chromatographic purifications severely affect the overall waste amount, thus a final *E* factor value of 3080 is obtained.

### 2.2 Chiral 4‐(dimethylamino)pyridines

**Figure 4:**
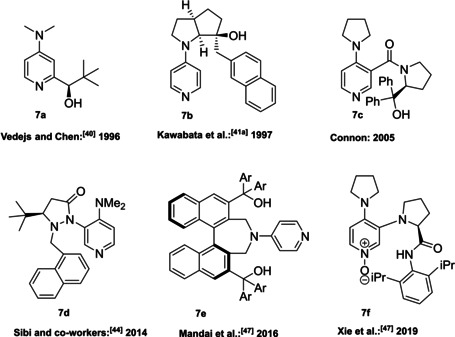
Selected examples of chiral DMAP **7 a**–**7 f**.^[46]^

4‐(Dimethylamino)pyridine (DMAP) and its derivatives are generally employed as acyl transfer catalysts^[39]^ and the pioneering paper of Vedejs and Chen paved the way to the organocatalyzed asymmetric version of these reactions.^[40]^ Nowadays, different chiral DMAPs are available and, as it can be seen from Figure 4, the induction of stereocontrol is linked to the presence of an element of chirality in the DMAP backbone (central, axial, planar, and helical chirality).[^41^, ^42^] A limitation in the employment of these catalysts is their acute dermal toxicity which can be reduced through DMAP salt formation, the salt produces, in fact, only local irritation. Alternatively, DMAP can be immobilized, allowing also for its recyclability thus increasing process sustainability.^[43]^

**Scheme 7:**
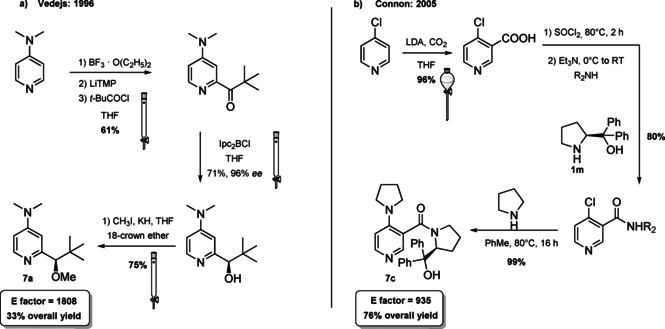
a) Synthetic protocol reported by Vedjes and Chen to obtain the first chiral DMAP 7a; b) Synthetic protocol reported by Connon to obtain bifunctional chiral DMAP 7c. *ee*=enantiomeric excess (LiTMP= lithium 2,2,6,6‐tetramethylpiperidide, LDA= lithium diisopropylamide, Ipc_2_BCl= −‐B‐chlorodiisopinocampheylborane).

The chiral DMAP **7 a**, developed by Vedejs and Chen, is a quite simple compound, in which a chiral center is present on a branched chain installed on the α‐position to the nitrogen in the pyridine ring (Figure 4 and Scheme 7a).^[40]^ A similar approach was later applied by Connon et al., installing a chiral amine, such as (*S*)‐α,α‐diphenylprolinol (**1 m**), through amide bond formation on the β‐position of the pyridine ring (compound **7 c**, Scheme 7b).^[41g]^ In this case, to induce remote stereocontrol the hydroxyl group is not protected and exploited for hydrogen‐bond formation. The synthetic procedures reported are based on a classical chemistry. As it can be seen from Scheme 7a, compound **7 a** can be obtained in four synthetic steps. Despite THF is employed as the solvent in all steps, sequential reactions are not telescoped in a one‐pot multistep reaction, but all intermediates are isolated through three column chromatographies. The employment of additional solvent contributes to an *E* factor of 1808. Connon et al. managed to reduce the number of purification steps, obtaining a lower *E* factor of 935; anyway, the employment of thionyl chloride and low temperature lithiation is not aligned with green chemistry principles (Scheme 7b).

**Scheme 8:**
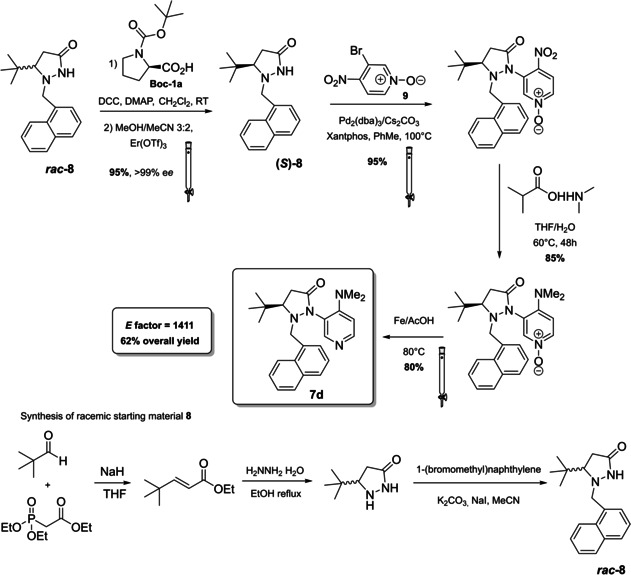
Synthetic procedure to obtain DMAP with fluxional chirality 7d (Pd2 (dba)3 = Tris(dibenzylideneacetone)dipalladium(0)).

Applied to the kinetic resolution of axially chiral biaryl compounds (selectivity factor 51) and secondary alcohols, DMAP with fluxional chirality **7 d** has been introduced by Sibi and co‐workers in 2014 (Figure 4 and Scheme 8).^[44]^ A fluxional group attached to the nitrogen atom of a chiral pyrazolidinone, whose steric hindrance can be modulated, is the main distinguishing peculiarity of these catalysts. The second nitrogen of the pyrazolidinone moiety is directly link to the *meta* position of the catalytic site embodied by a DMAP derivative (Scheme 8). Eight synthetic steps are necessary to construct fluctional catalyst **7 d** starting from a simple aldehyde. In order to build just the chiral pyrazolidinone **8**, five out of eight steps are employed. In fact, **8** is prepared as racemic mixture and then the single enantiomer is resolved. Despite the natural compound **1 a** is employed as the resolution agent, this strategy goes against the 8^th^ principle. In fact, **1 a** should be priorly protected with tert‐butyloxycarbonyl (Boc) group ; then, the resulting protected scaffold should be installed on the pyrazolidinone to obtain a diastereomeric pair, from which (*S*)‐enantiomer of **8** can be separated by chromatography. In order to introduce the DMAP structure, chiral **8** is subjected to a coupling reaction in the presence of 3‐bromo‐4‐nitro‐pyridine N‐oxide (**9**). The nitro group is then exploited to insert the 4‐dimethylamino functionality. Catalyst **7 d** is finally obtained treating the *N*‐oxide with iron powder in acetic acid at 80 °C. Apart from CH_2_Cl_2_ and THF, all the solvents employed have been classified as medium issue solvents.^[45]^ For a potential scale‐up of this reaction on industrial scale, one should take into account the presence of potential hazards derived from the employment of flammable liquids and solids and the toxicity against aquatic life of compounds like *N*,*N’*‐dicyclohexylcarbodiimide (DCC) and **7 d** itself. This process is characterized by an *E* factor of 1411.

**Scheme 9:**
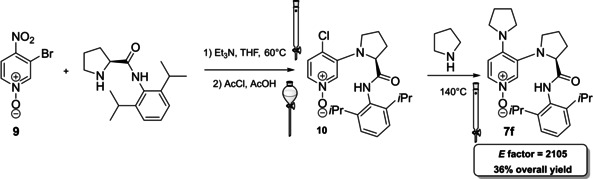
Chiral DMAP−N‐oxide 7 f synthesis.

If Sibi et al.^[44]^ employed N‐oxide as intermediates, recently Xie et al. applied chiral DMAP‐N‐oxides **7 f** as acyl transfer catalysts for Steglich rearrangement (Figure 4 and Scheme 9).^[46]^ The approach applied is very similar to the one of Connon, in fact the catalyst exploits an amide group as hydrogen‐bond donor and the simple pyridine is replaced by an N‐oxide (Scheme 9). Starting from the commercially available **9**, the reaction can be performed on a gram scale. Although it accounts only for three steps, the *E* factor is quite high (2105), since each intermediate is purified. This could be, mainly, due to the authors intent to characterize all the intermediates. For what concerns N‐oxides, no information about safety has been reported. Hazards about the other reagents employed have been discussed in other sections.

**Scheme 10:**
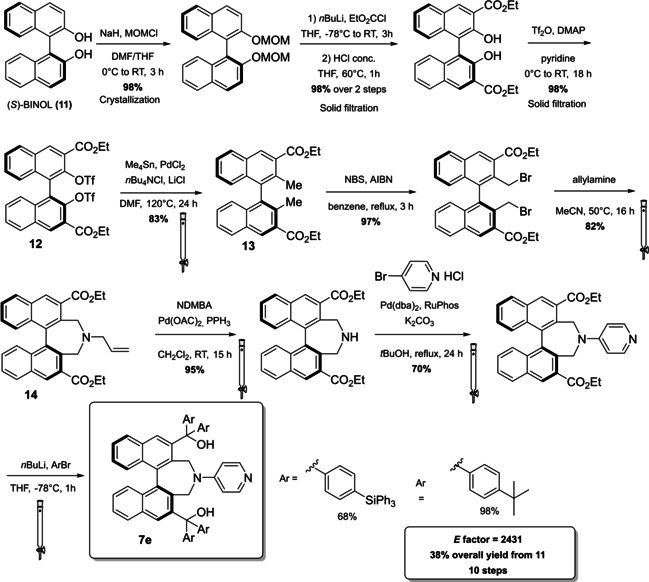
Axially chiral DMAP 7 e derived from (*S*)‐BINOL 11 (MOMCl= Methoxymethyl chloride, NBS= *N*‐bromosuccinimide, AIBN= α,α,′‐azoisobutyronitrile, NDMBA=1,3‐dimethylbarbituric acid).

As last example for this section, we analyze the synthesis of the axially chiral DMAP **7 e** derived from (*S*)‐BINOL (**11**) in 10 steps with an overall yield of 38 % (Scheme 10).^[47]^ Employing well established protocols a binaphthyl unit, assuring chirality, is inserted at the C_4_ position of the pyridine ring. At the same time, the pyridine activity is enhanced by the lack of substituents at its C_2_ and C_3_. The catalyst backbone is constructed starting from (*S*)‐BINOL **11** (410 g) applying a series of lithiation and metal‐catalyzed cross couplings. As emphasized by the authors, the conversion of ditriflate **12** to compound **13** is realized through a Migita–Kosugi–Stille coupling. This reaction employs Pd nanoparticles as catalyst avoiding the use of Me_2_Zn, which being pyrophoric and costly could represent a problem for large scale production. The bromination is realized with *N*‐bromosuccinimide and the bromine atoms are then exploited to close a seven‐member ring including an amine group after deprotection (intermediate **14**). Pyridine can be introduced applying the Buchwald–Hartwig amination. When possible, crystallization and solid filtrations are preferred to chromatography: in fact, only four column chromatography are realized along the ten synthetic steps. The catalyst obtained is very reactive and only 0.5 mol % are sufficient to realize the kinetic resolution and desymmetrization of diols in quantitative yield and high enantiomeric excess. All the reaction medium employed with the exception of *t*‐BuOH and MeCN are classified as major issues solvents.

## 3 Brønsted Base Catalysts

### 3.1 Organosuperbases: Chiral Guanidines and Iminophosphoranes

Chiral guanidines and iminophosphoranes can be considered both as Brønsted bases, according to the classification of List and Maruoka, and as organosuperbases.^[6]^ They are, in fact, characterized by a strong basicity which confers them the ability to deprotonate and activate weakly acidic pronucleophiles.^[48]^ A very recent review analyzes the basicity of these compounds and their application as organocatalysts.^[49]^ For this reason, this chapter will focus on organosuperbase structures and synthesis with an emphasis on the respect of the green chemistry principles.

#### 3.1.1 Chiral Guanidines

**Figure 5:**
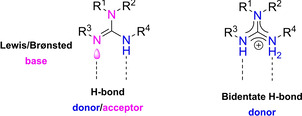
General structure of guanidines emphasizing the donor/acceptor ability of this moiety.

The classification of chiral guanidines as organo‐superbases is linked to the combination of strong basicity with hydrogen‐bond donor ability (Figure 5) resulting from the formation, after protonation, of a high delocalized conjugated guanidinium system. In order to become widely exploited as organocatalysts, their development had to face different challenges. In fact, there was a general lack of methodologies giving access to guanidine moiety, it was very difficult to introduce chiral groups around the guanidine and these compounds resulted very difficult to isolate because of their intrinsic basicity and polarity.

**Figure 6:**
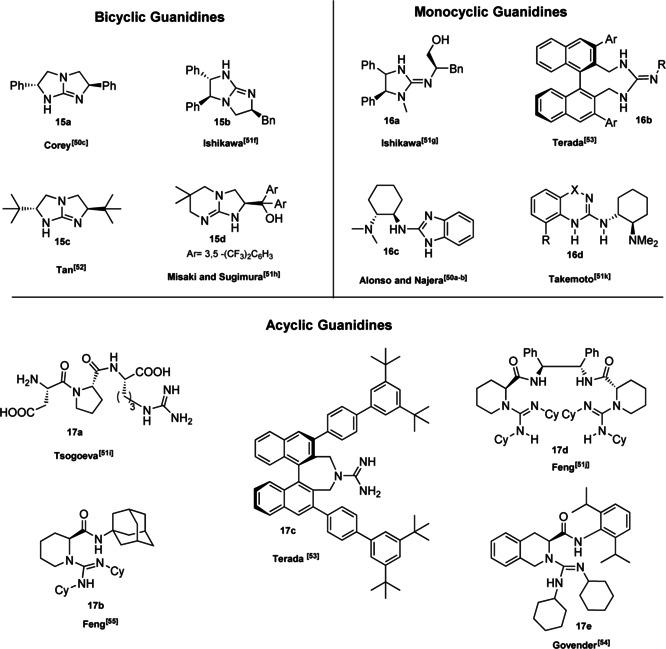
Selected examples of chiral guanidine superbases.

Pioneering studies were realized by the groups of Nájera^[50a]^ and Davis^[50b]^ in the 1990s, but we had to wait the end of the century to have a breakthrough. Corey and Grogan^[50c]^ and the group of Lipton^[32a]^ worked extensively in order to make the synthesis of chiral guanidines more accessible and thus exploiting these compounds as effective chiral organocatalysts. Figure 6 shows a selection of chiral guanidine organocatalysts which are classified as bicyclic, monocyclic and acyclic compounds, according to the inclusion or not of the guanidine moiety in a cyclic framework. In general, bicyclic guanidines **15 a**–**15 d** are characterized by a rigid structure influencing their chemical, physical and electronic properties. Most of monocyclic guanidines **16 a**–**16 d** have a pseudo‐C_2_‐symmetry, while the open chain compounds **17 a**–**17 e** are sterically more easily accessible, more flexible and their conformational freedom can be reduced introducing hydrogen‐bond donor groups or sites for substrate–catalyst interactions.

**Scheme 11:**
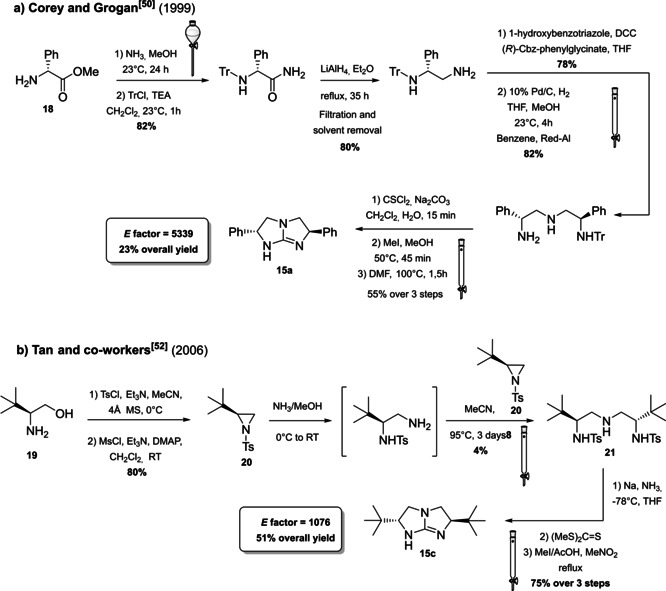
Comparison between Corey and Tan methodologies to obtain bicyclic chiral guanidines 15a and 15c (Tr= triphenylmethyl chloride, TEA= triethylamine).

The development and the application of these compounds and their derivatives in asymmetric organocatalyzed reactions has already been critically addressed in several comprehensive reviews and will not be the subject of this section.^[51]^ In contrast, we will focus on selected methodology to access chiral guanidine organocatalysts. The first general route to chiral C_2_‐symmetric bicyclic guanidines without the need for resolution was reported by Corey and Grogan employing the chiral (*R*)‐methylphenylglycinate (**18**) as starting material (Scheme 11a).^[50]^ The reaction scheme proposed by Corey and Grogan is composed by eight steps in which each reaction can be realized on gram scale and the guanidine product **15 a** is obtained in 23 % overall yield. The process is characterized by an *E* factor of 5339, a high value that takes into account several purifications by column chromatography and the employment of stoichiometric co‐reagents. Moreover, some toxic reagents such as iodomethane and thiophosgene are employed. A more performing and efficient approach was proposed by the Tan group in 2006 (Scheme 11b).^[52]^ It is, in fact, composed only by four synthetic steps, an increased overall yield of 50 % and an *E* factor value of 1076 which is almost five times smaller than the one obtained by Corey and Grogan. The key feature of this synthetic methodology is the employment of aziridine intermediate **20**, which can be easily obtained from commercially available amino alcohols such as *L*‐*t*‐leucinol (**19**). *N*‐tosyl aziridine (**20**) can participate in a regio‐ and stereospecific ring opening, giving access to a triamine **21**, which, after removal of tosyl groups, can be cyclized in a one‐step one‐pot process to the corresponding guanidine **15 c**. The cyclization is still realized in the presence of toxic thiophosgene and iodomethane, but more attention is given to the prevention of waste limiting intermediate purification. Contrary to Corey and Grogan, the procedure of Tan and co‐workers includes some cryogenic reactions that compromise the energy efficiency of the process.

**Scheme 12:**
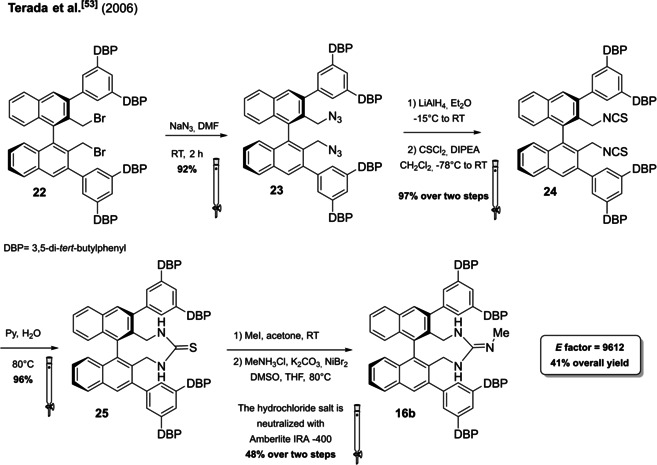
Synthetic steps for the production of an axially chiral guanidine 16b.

Monocyclic guanidines are frequently included in a C_2_‐symmetric framework in which the stereocontrol is obtained by the introduction of bulky groups around the guanidine moiety (Figure 6). This can be easily obtained including the guanidine inside a cycle which is constructed on an axially chiral BINOL skeleton (Figure 6 compound **16 b**). In fact, the bulky aryl substituents at the 3,3’‐positions are able to break the symmetry, creating an effective chiral environment around the guanidine. Terada group worked extensively on these structures which can be obtained by lengthy synthetic protocols composed by multiple steps. The efforts needed to obtain these structures are balanced out by the fact that monocyclic guanidines are high active and selective catalysts in very low loading. Moreover, the guanidine catalyst can be recovered from the reaction mixture as hydrochloride salt applying a simple extractive workup. As a representative of this class of compounds the synthesis of one of Terada catalyst is presented.^[53]^ Compound **16 b** can be obtained from (*R*)‐BINOL **11** in around 20 % overall yield by a synthetic protocol composed by thirteen steps (Scheme 12). As it can be seen from Scheme 12 only the last six steps are analyzed in this section since the construction of BINOL skeleton will be addressed in Chapter 4. The introduction of the monocyclic guanidine moiety is realized by nucleophilic substitution of the dibromide BINOL‐derivative **22** with sodium azide in dimethylformamide as solvent. The reduction of the chiral diazide **23** with LiAlH_4_ allows the introduction of a diamine functionality, which is then converted to a diisothiocyanate **24**. Once the diisothiocyanate has been installed, the monocycle can be closed exploiting thiourea formation. In the last two steps the thiourea **25** is converted into the monocyclic guanidine. The synthetic protocol is very robust: in fact, almost each step is characterized by a yield above 90 %, but almost all intermediates are purified by column chromatography.^[53]^ The latter which is responsible for around 40 % of total waste mass, contributes to a very high *E* factor of 9612 only for the last six synthetic steps. Moreover, no specific attention is paid to the use of safer solvents and reagents (5^th^ principle) and to energy efficiency (6^th^ principle), in fact most of the reactions require low temperatures (from −78 °C to −15 °C). The principle of atom economy is respected.

**Scheme 13:**
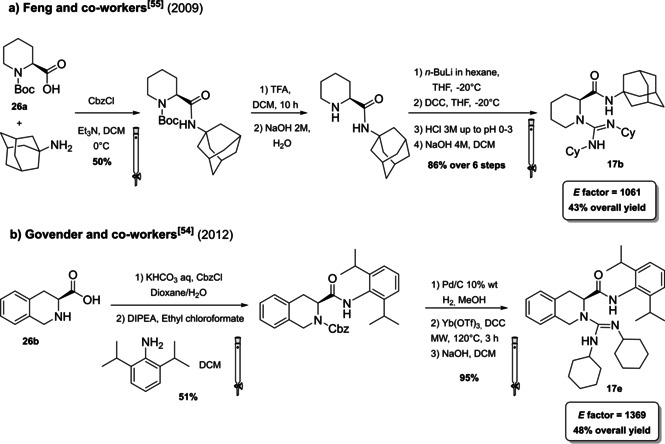
Feng and Govender synthetic procedures to obtain chiral open‐chain guanidines 17b and 17e.

The open‐chain guanidines **17 a**–**17 e** are more easily accessible, but generally characterized by a lower enantioselectivity. Two examples from the Govender^[54]^ and Feng^[55]^ groups have been selected. In general, the acyclic guanidine is obtained through a multistep synthesis starting from β‐amino carboxylic acids **26 a** or **26 b** on which the amine functionality is exploited to install the guanidine unit, while the carboxylic acid is converted to an amide bringing a bulky substituent (Scheme 13). In order to introduce the amide functionality, the secondary amine group is protected through Cbz or Boc and the protected group is removed before guanidine introduction (against 8^th^ principle). In both cases, two chromatographic purifications are required. The procedures reported by the groups of Feng and Govender are comparable and, in terms of *E* factor, the synthetic route of Feng and co‐workers (*E* factor 1061) should be preferable compared to the Govender protocol (*E* factor 1369). Anyway, less hazardous reagents were applied by the Govender group. In addition to protecting groups, Feng et al., in fact, employed trifluoroacetic acid (TFA), which is corrosive and *n*‐BuLi which is flammable and can ignite spontaneously in the presence of water. The employment of *n*‐BuLi not only implies to work in dry reaction conditions but it also needs for low reaction temperature. A solution to this issue would be to run this reaction in a flow chemistry fashion.

#### 3.1.2 Iminophosphoranes

**Figure 7:**
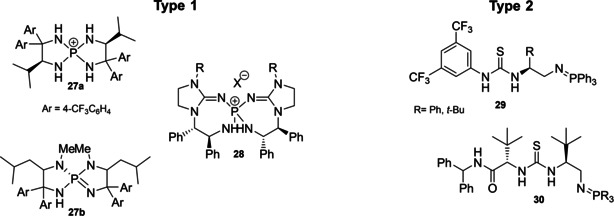
Classification of chiral iminophosphoranes.

Iminophosphoranes belong to the family of chiral organosuperbases and they can be classified in two main groups (Figure 7). Type 1 iminophosphoranes **27** and **28** are characterized by a chiral spirocyclic system containing a chiral phosphorous atom. Type 2 iminophosphoranes **29** and **30** bear an acyclic system in which a hydrogen‐bond donor group is included, making these compounds bifunctional catalysts.^[56]^

**Scheme 14:**
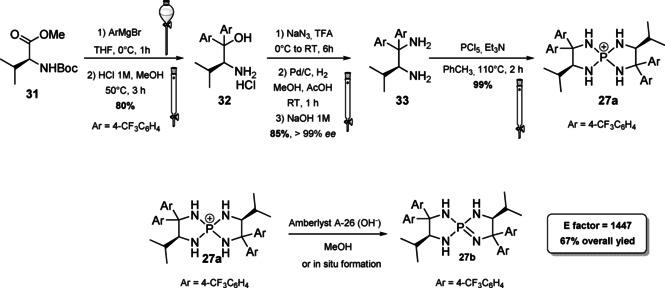
Reaction scheme for the synthesis of [5,5]‐*P*‐spirocyclic iminophosphoranes of Type I.

In this section, a brief overview on iminophosphoranes will be given analyzing the synthetic protocols of one compound for each type. For Type I iminophosphoranes, we report the synthesis of the [5,5]‐*P*‐spirocyclic scaffolds **27 a** and **27 b**, which were introduced by Ooi and co‐workers in 2007 (Scheme 14).^[57]^ Modulating the structure of starting material and Grignard reagent it is possible to access a library of compounds. The synthesis of the salt requires the preparation of the corresponding 1,2‐diamine **33**, which can be obtained from a sequence of five steps starting from Boc‐*L*‐Val‐OMe (**31**). This amino‐acid derivative (7^th^ principle) is converted to a tertiary alcohol by reaction with a Grignard reagent followed by acidification. The amino alcohol **32** is then treated with NaN_3_, and the azide functionality introduced by nucleophilic substitution is reduced in the presence of Pd/C and H_2_ to the corresponding diamine **33**. Iminophosphoranes **27 a** are released upon treatment with phosphorus pentachloride in the presence of Et_3_N as HCl scavenger. Salts of **27 a** are stable and can be isolated by column chromatography. In order to obtain free iminophosphorane **27 b**, the salt has to be treated with Amberlyst A‐26 or activated in situ by addition of a base (*t‐*BuOK). The reaction can be run on gram scale starting from amino acid derivative **31**. The yield is moderate (around 67 %) and the *E* factor is 1447.

**Scheme 15:**
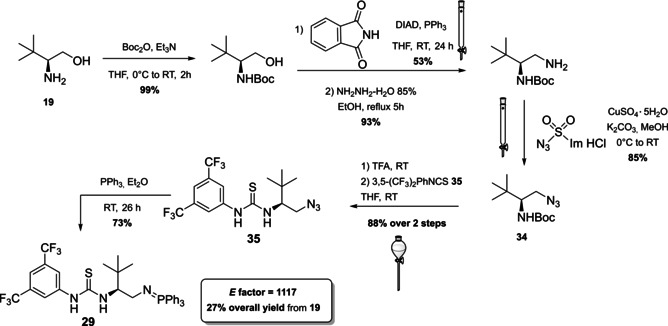
Synthetic protocol to access bifunctional iminophosphorane 29 (DIAD= diisopropyl azodicarboxylate).

Safety concerns arise from the employment of hazardous chemicals such as sodium azide and phosphorus pentachloride (against 3^rd^ and 4^th^ principles).^[57]^ Type 2 iminophosphorane catalysts have been introduced by the group of Dixon in 2013 and allow the dual activation of the substrate through hydrogen bonds (Scheme 15).^[58]^ The authors idea was to insert the Brønsted basic moiety on a structure containing a hydrogen‐bond donor, such as a thiourea, in the last step of the synthetic protocol. This could be realized applying the Staudinger reaction on a preformed chiral organoazide **35**. *L*‐*tert‐*leucine‐derived azide **34** can be obtained on gram scale starting from *L*‐*t*‐leucinol (**19**), the amino alcohol derived from *t‐*leucine (7^th^ principle, Scheme 15). The strategy applied relies on primary amine protection (against 8^th^ principle) followed by reaction with phthalimide, a known ammonia synthetic equivalent which, upon cleavage, allows the introduction of a second amine functionality. According to the 3^rd^ and 4^th^ principles ammonia, which is corrosive and toxic for aquatic life, is replaced by phthalimide, for which no hazards have been reported by ECHA.^[59]^ Nevertheless, the cleavage needs the application of hydrazinium oxide, which is suspected to cause cancer and, if released in the environment, is very toxic to aquatic life with long lasting effects. Then, an azide group is introduced applying the diazotransfer reagent imidazole‐1‐sulfonyl azide hydrochloride, a bench stable and crystalline equivalent of trifyl azide (Scheme 15).^[60]^ The amine group is then deprotected and the free primary amine is exploited to insert the thiourea moiety. In the late stage, the Staudinger reaction with triphenylphosphine allows the isolation by precipitation and filtration of the bench‐stable bifunctional iminophosphoranes **29**. The calculated *E* factor starting from **19** is 1117. A particular attention is given to prevention of waste (1^st^ principle) considering that a six steps protocol counts only two purifications by column chromatography. Moreover, in the point of view of green chemistry, some iminophosphorane catalysts have been immobilized on a solid support allowing catalysts recovery up to eleven times without affecting reactivity.^[61]^

**Scheme 16:**
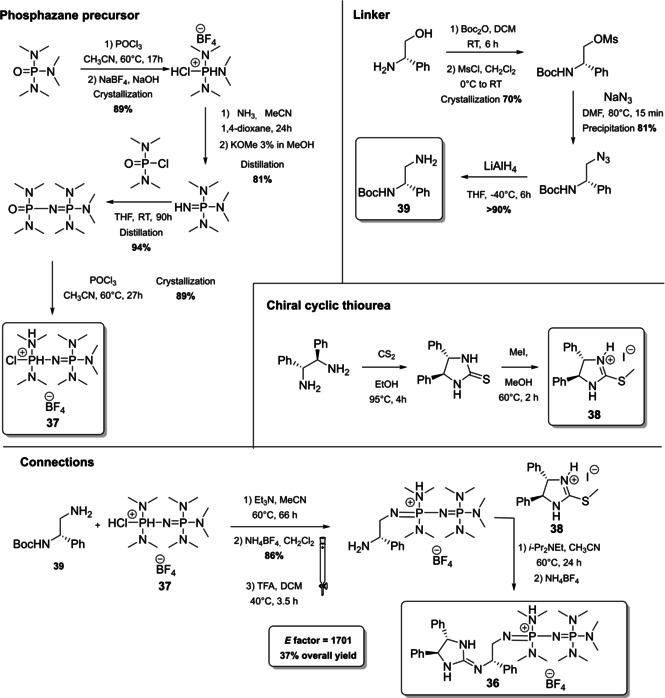
Synthesis of chiral cooperative binary base catalyst 36.

The last catalyst we present has been developed very recently by the group of Terada, and it represents the first example of “chiral cooperative binary based catalyst” **36**, a compound able to activate less acidic pronucleophiles such as α‐phenylthioacetates (Scheme 16).^[62]^ Catalyst **36** presents, in fact, two organosuperbase functionalities, with a different role in the catalytic process, one acting as organosuperbase, the other operating substrate recognition. A convergent synthesis is exploited to obtain compound **36** starting from a *P*_2_‐phosphazene precursor **37** (it will act as organosuperbase site in the catalyst), a chiral cyclic thiourea **38** (it will act as substrate recognition site) and a chiral 1,2‐diaminoethane derivative **39** (linker between the two sites). If one considers the synthetic steps needed to obtain all the precursors and the one necessary to connect them (overall seventeen steps), the *E* factor obtained is very low (1701) in comparison with other protocols composed by a similar number of steps. A particular attention is, in fact, dedicated to the prevention of waste (1^st^ principle) avoiding all the unnecessary intermediate purifications and preferring distillation or crystallization to column chromatography. On the other hand, standard reagents are employed and most of them pose severe concerns about safety (as examples: NaBF_4_, KOMe, CS_2_, Et_3_N), human health (as examples: POCl_3_, MsCl, NaN_3_, CS_2_, MeI, Et_3_N) and environmental health (as examples: NH_3_, NaN_3_, MeI, TFA).

### 3.2 Cinchona alkaloids, 9‐amino (9‐deoxy)*epi* Cinchona alkaloids, and their derivatives

**Figure 8:**
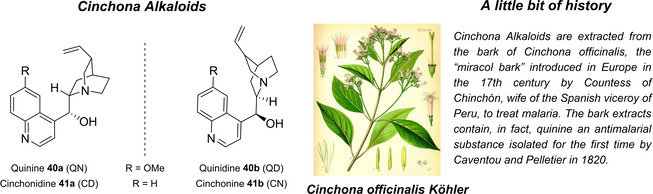
Cinchona alkaloids pseudo‐enantiomers and a bit of history on the discovery of these compounds.^[64]^

Cinchona alkaloids are naturally occurring molecules (Figure 8), which can be considered as the roots of organocatalysis. In fact, at the beginning of the 20th century Bredig and Fiske were the first to describe the employment of the naturally occurring molecules quinine as catalyst.^[63a]^ This innovative concept had to wait for the Wynberg studies in 1975^[63b]^ to be recognized and further for the early 2000s to blossom.[^7b^, ^7c^] After that, the gold rush in organocatalysis started.[^11b^, ^11c^]

**Scheme 17:**
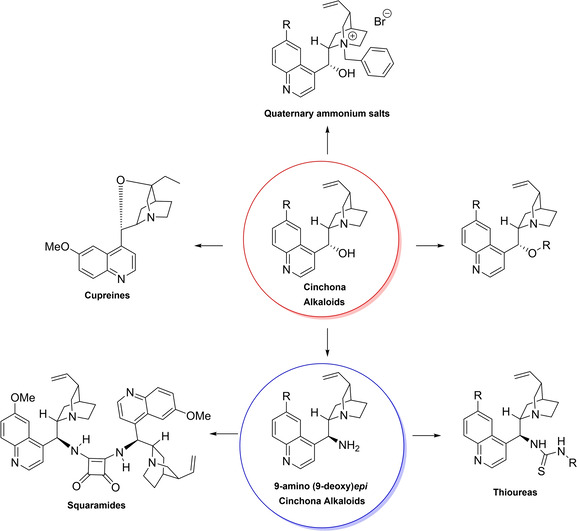
Variety of catalysts that can be obtained from Cinchona alkaloids.

Their success as organocatalysts is mainly linked to their conformational flexibility in solution, which can be tuned by different stimuli, affecting the catalytic behavior and allowing these compounds to have high tolerance towards substrates.^[65]^ Moreover, the OH group at the C9 atom can be exploited to form hydrogen bonds, which, in synergy with the quinuclidine nitrogen, makes the alkaloid a bifunctional catalyst. Being natural compounds, Cinchona alkaloids are not only the ideal catalysts to meet the green chemistry principles, but they can also be considered a molecular platform on which to construct different families of catalysts including phase‐transfer catalysts (Scheme 17). Between them, 9‐amino (9‐deoxy)*epi* Cinchona alkaloids have a central role. These catalysts have been introduced in early 2007, by the groups of Chen^[66]^ and Melchiorre^[67]^ and McCooey and Connon,^[68]^ who were working independently and almost at the same time on this topic. These compounds have allowed to expand the substrate scope of aminocatalysis to ketones, α,β‐unsaturated compounds, and α‐branched aldehydes, keeping high levels of stereocontrol and exploiting different activation modes (enamine, dienamine, trienamine, iminium ion).^[65]^ The introduction of the primary amine functionality, in fact, gives to the Cinchona alkaloid an additional chemical handle for covalent interactions. The quinuclidine nitrogen maintains the role of base participating in nucleophile/electrophile activation.

**Scheme 18:**
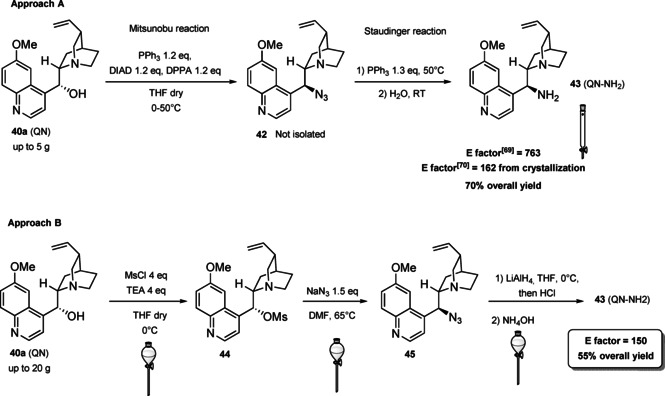
Different approaches for the synthesis of **43**.

Now our discussion will focus on the synthesis of 9‐amino (9‐deoxy)*epi* Cinchona alkaloids such as compound **43** (Scheme 18). The general procedures described for quinine (**40 a**) can be applied to all the other members of the Cinchona alkaloids family. Two different approaches can be pursued:[^69^, ^70^]

- the amination of alcohol at the C9 atom according to Mitsunobu protocol followed by in situ azide Staudinger reduction (Scheme 18, Approach A); and
- mesylation at the C9 atom followed by nucleophilic displacement with sodium azide and reduction with lithium aluminium hydride (Scheme 18, Approach B).

In both cases, the reaction proceeds with inversion of configuration at the C9 atom. Approach A (Scheme 18) is based on a one‐pot two steps protocol characterized by mild reaction conditions and high stereoselectivity. The protocol is compatible with the presence of other functional groups on the starting material and can be scaled up to five grams of alkaloid. In the Mitsunobu reaction, the alcohol at the C9 atom is reacted with a nucleophile in the presence of triphenylphosphine (PPh_3_) and diisopropyl azodicarboxylate (DIAD). In order to introduce an amine functionality, the nucleophile employed is diphenylphosphoryl azide (DPPA)_,_ a safer alternative to hydrazoic acid. Azide **42** is not isolated but reduced in situ adding an excess of PPh_3_. A consecutive hydrolysis according to Staudinger reaction affords product **43**. In accordance with green chemistry, all the sequence of reactions is telescoped into a single solvent in a one‐pot fashion. If 9‐amino (9‐deoxy)*epi* quinine (**43**) is isolated by column chromatography, the process *E* factor is 763.^[69]^ With regard to waste prevention, column chromatography can be avoided treating the crude reaction mixture with aqueous HCl solution. The addition of an acid causes the precipitation of product hydro‐chloride salt. The free amine can be, later, obtained by neutralization with NH_4_OH aqueous solution.^[70]^ Notably, the removal of chromatographic purification makes the protocol greener with an *E* factor which is 4.7 times smaller (162 vs 763) than the previous including column on silica gel.

Despite an *E* factor of 162, Melchiorre and co‐workers pointed out that approach A has some limitations for large‐scale production, the major concerns being the employment of expensive reagents (DIAD and DPPA) and of an excess of PPh_3_. Indeed, high amounts of triphenylphosphine oxide are yielded as waste causing difficulties during purification. The alternative protocol proposed by Melchiorre and co‐workers (Scheme 18 Approach B)^[70]^ is composed by three steps, in which each product is purified by an extractive workup avoiding the production of silica gel waste in accordance with the 1^st^ principle. The protocol appears more economic, in fact the expensive DIAD and DPPA are replaced by methanesulfonyl chloride (MsCl) and NaN_3_. On the other hand, approach B is not in line with the 3^rd^ and the 4^th^ principles (safer synthesis and safer chemicals). In fact, sodium azide is more toxic than DPPA, the equipment to make the nucleophilic displacement should be free of heavy metals, in order to avoid detonation in case of thermal or mechanical stress, and the waste should be handled with care, in order to avoid the formation of the poisonous and explosive HN_3_. Interestingly, the azides derived by Cinchona alkaloids are not a problem for safety since their exothermic degradation starts more than 60 °C above the reaction temperature. The last step is the reduction of **45** which can be realized with LiAlH_4_ or H_2_/Pd. The latter reduction methodology is applied to obtain the simultaneous reduction of either the azide and the double bond, to yield the dihydro derivative. Also in this case, the product is purified by hydro‐chloride salt formation. As emphasized by Melchiorre and co‐workers, the salt is an air stable solid that can be stored for weeks and when needed neutralized to release the free amine. A lower *E* factor of 150 was found for approach B, even though **43** is obtained in a lower yield (55 % yield vs 70 % yield for approach A).

## 4 Brønsted Acid Catalysts

### 4.1 Phosphoric acids

**Figure 9:**
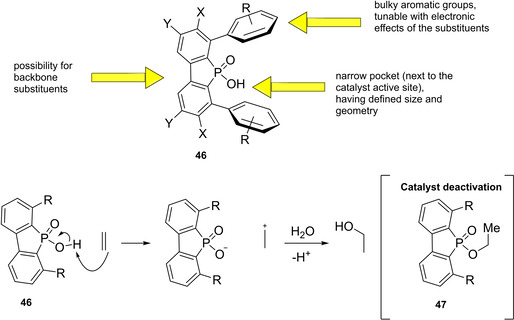
General structure of Cornforth phosphinic acids and proposed mechanism for phosphinic acid‐catalyzed hydration of alkenes.

Almost the entire class of Brønsted acid organocatalysts consists of very complex scaffolds. The reason for this is due to the mutual interconnection, for historical reasons, of the concepts of Brønsted acid organocatalyst and confined catalyst. In fact, the first examples of Brønsted acid organocatalysts, namely axially chiral BINOL‐derived phosphoric acids, take the moves from the molecular scaffold of Sir John Cornforth's phosphinic acids, dating back to 1978.^[71]^ Cornforth, winner of the Nobel Prize for Chemistry in 1975 for his contributions to the stereochemistry of enzyme catalyzed reactions, was the first to apply this know how in designing the structure of a catalyst for hydration of C−C double bonds. In particular, he guessed the potential of introducing bulky aromatic groups in appropriate positions of the phosphinic acid scaffold **46**, in order to obtain a narrow pocket for substrates, mimicking the active site of an enzyme. Furthermore, electron‐donating or withdrawing substituents on these aryl groups have the function of tuning the acidity of the compound, apart from defining the pocket size and geometry (Figure 9).

The work of Cornforth deeply influenced the pioneering publications of Akiyama et al.^[72]^ and Uraguchi and Terada^[73]^ in the field of chiral phosphoric acids. For what concerns phosphoric acid organocatalysts themselves, regardless of their structure, according to List and Maruoka definition,^[9]^ there is no unequivocal criterion to classify them as Brønsted acids rather than Lewis acids. This ambiguity is due to the strong dependence of their activation mechanism on the substrates, the reactants and the reaction conditions. Thus, it is possible to distinguish among protonation/ion pairing and coordination/hydrogen bond donation,^[74]^ and even between mono‐ and bifunctional modes of activation.^[75]^ This formal classification is not always easy to verify from an experimental point of view. However, mechanistic insights are not the subject of this review.[^74^, ^75^] For simplicity, all phosphoric acid organocatalysts, because of their chemical nature of phosphoric acid cyclic diesters (p*K*_a_ between 12 and 14 in acetonitrile),^[76]^ will be classified here as Brønsted acids. In the following sections phosphoric acids catalysts will be analyzed according to their backbone.

#### 4.1.1 BINOL‐based phosphoric acids (BPAs)

**Scheme 19:**
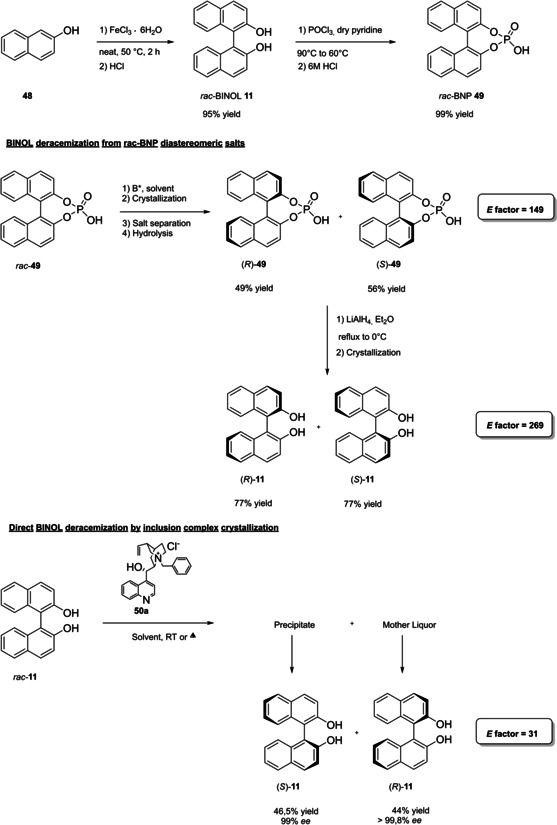
The most common complete synthetic routes to enantiopure BINOL **11**.

In 2004, the first examples of BINOL‐PAs appeared in the literature by Akiyama et al.^[72]^ and Uraguchi and Terada^[73]^ as stereoselective catalysts for Mannich‐type reactions. From that moment, a wide variety of highly performing BINOL‐PAs started to appear in the literature. One of the most popular phosphoric acid organocatalysts^[77]^ is 3,3′‐bis(2,4,6‐triisopropylphenyl)‐1,1′‐binaphthyl‐2,2′‐diyl PA, known as TRIP, developed by List and co‐workers.^[78]^ The very first common structural feature of these catalysts is obviously (1,1′‐binaphthalene)‐2,2′‐diol (BINOL) backbone. BINOL **11** itself is a synthetic scaffold, whose large availability on the market in both enantiomers at reasonable price is justified by the full‐blown role of its derivatives as “privileged chiral catalysts”.^[79]^ However, neither this molecule, nor its direct precursor are derived from natural sources. Indeed, historical synthetic route to **11** includes Fe^III^‐catalyzed radical coupling of 2 equivalents of β‐naphthol (**48**),^[80]^ followed by an articulate racemate resolution (Scheme 19). The synthesis starts from **48**, which although not derived from natural feedstocks, can be assumed on edge a low‐environmental impact synthetic molecule, being obtainable on industrial scale by air oxidation of naphthalene in steam current. In this case, no waste mass is assumed to derive from the process, since the oxygen in the air is a renewable source and steam plays a role in the water cycle. Historical preparation from naphthalene sulfonation/cleavage in molten alkali,^[81]^ as well as other inconvenient chemical processes (including non‐regioselective procedures) will then be neglected. The homocoupling of **48** to *rac*‐**11** requires over‐stoichiometric amount of the iron hydrate salt. Nevertheless, the product can be afforded in excellent yield by gentle heating in neat conditions. This feature makes the protocol preferable with respect to other available procedures based on catalytic copper,^[82]^ vanadium,^[83]^ ruthenium,^[84]^ rhenium,^[85]^ solid supports like alumina[^82e^, ^82f^, ^82g^, ^82h^] or acidic silica,^[86]^ electrochemical methods^[87]^ and even iron chloride hexahydrate under microwave irradiation.^[88]^ Benefits of employing catalytic amounts of the metal species are compensated by the detrimental use of volatile organic compounds (VOCs) such as DCM or petroleum ether, scarce eco‐compatibility of the exhausted catalyst, low energy efficiency in terms of the 6^th^ principle and difficulties in the scaleup. In fact, iron chloride is perfectly eco‐compatible (being a component of lava rocks), non‐volatile and cheap, and heating does not represent a problem on an industrial scale. At this point the resolution of the racemates has to be performed, and the general route proposed in Scheme 19, which is a common chemical resolution, is not the only valid alternative. If the above depicted strategy is followed, 1,1’‐bi‐2‐naphthyl‐phosphate (BNP) **49** has to be prepared, according to the general protocol of direct reaction with POCl_3_ by Jacques et al.^[89]^ It is worth noticing that, although the reaction is critically performed in dry pyridine as the solvent, its amount is relatively limited, and no further chromatographic purification is required, but simple precipitation and washing with diluted HCl currently makes it the optimal strategy. The resolution of the two enantiomers can then be performed with cinchonine (**50 a**) (see Scheme 19 for yields). Other chiral amines or auxiliaries can be instead employed, this item affecting both the cost and the *E* factor of the process. In fact, inexpensive 2‐aminobutanol^[90]^ and more complex amines lead to lower yields and enantioselectivities. At this point, the very first and simplest chiral BINOL‐derived phosphoric acid has been afforded in enantiopure form. To be fair, simple **49** was often employed as a chemical resolving agent^[89]^ rather than as a chiral catalyst. The *E* factor for enantiopure **49**, resulting from the first four steps proposed in Scheme 19 and considering the masses of both the isolated enantiomers as useful products, is 149 if cinchonidine (**41 a**) is employed as the chiral base.^[91]^ This value could be subject to decrease, considering the recycling of solvents and resolving agent **41 a**. Further dephosphorylation with lithium aluminum hydride finally affords the atropoisomers of **11**, each one in 77 % yield. Because of this additional step, the *E* factor rises to 269 for **11**, considering the masses of both the enantiomers as useful products also in this case. Different strategies to resolve the racemic mixture of the atropoisomers are described in various reviews specifically dedicated to the synthesis of BINOL **11**.^[92]^ However, since the aim of this review is to focus the attention on the greenness of organocatalysts synthesis, it is worth pointing out some important observations before proceeding with the discussion. In the above‐described historical route to access enantiopure BINOL, multiple derivatization steps affect the atom economy of the process and, consequently, the *E* factor. Similarly, the methods based on derivatization and protecting groups removal go against the 2^nd^ and the 8^th^ principle. Second, enzymatic resolution, even though it may appear a cheap alternative to chemical resolution when low‐cost enzymes (e. g., lipases from bacteria) are suitable for this purpose, it accounts for the biomass wasted in the enzyme isolation which affects the *E* factor and the atom economy of the overall process. Third, even if the product is afforded in high yield, the enantiopurity is not sufficiently high (*ee*<95 %), making necessary an additional crystallization which produces additional waste, while increasing the *E* factor at the same time. Therefore, only methods affording enntiopure **11** in yield and *ee* superior to 90 % will be analyzed.

These include examples of chemical resolution having improved *E* factor with respect to the route depicted in Scheme 19, namely racemate desimmetrization and asymmetric synthesis. Established that derivatization should be avoided, the best way to resolve racemic BINOL is obviously to directly proceed on this substrate by using chiral auxiliaries. *N*‐benzylcinchonidinium chloride (**50 a**) guarantees high yield and *ee* for both the enantiomers of BINOL and,^[93]^ despite its synthesis has to be taken into account (see Chapter 5), leads to a considerably lower *E* factor of 31, performing the process in refluxing acetonitrile as the crystallization solvent. The complementary approach with pseudoenantiomer **50 a** ^[94]^ affords the association complex of the (*R*)‐enantiomer as a precipitate. Employment of *trans‐*1,2‐ciclohexyldiamine,^[95]^ phenylethylamine,^[96]^ proline and prolinamide,^[97]^ 3‐alkyl‐4‐(1’‐phenylethylamino)butanoic acid,^[98]^ and other chiral amines^[99]^ or auxiliaries^[100]^ results in overall worse results in terms of yield or *ee*. Nevertheless, resolution strategies are limited by a maximum yield of 50 % for each enantiomer, even if both of them can be considered useful products. Deracemization strategies rely instead on the possibility of converting one enantiomer into the other, starting from the racemate, affording a single enantiopure useful product. An interesting mild deracemization protocol based on room temperature treatment with chiral copper complexes has been proposed by Wulff and co‐workers.^[101]^ The initial procedure was limited to the obtainment of (*S*)‐**11** in 94 % yield and 92 % *ee* (97 % yield, 92 % *ee* at −20 °C) with CuCl complex and (−)‐sparteine (**51**) on a 200 mg scale, while less efficient performances in terms of *ee* were obtained on a 5 g scale (98 % yield, 87 % *ee* at −20 °C) or employing corresponding copper(II) complex (94 % yield, 80 % *ee*).^[101a]^ Later in‐depth studies lead to the comprehension of the nature of deracemization process, which involves oxidation of the biaryl scaffold. The protocol was then improved by reducing the total amount of costly (−)‐sparteine, which was replaced by quenching with HCl (97 % yield, 92 % *ee*) or NaHCO_3_ (95 % yield, 93 % *ee*) and finally performed with O'Brien's diamine (synthetic analogue of non‐natural (+)‐sparteine) to obtain (*R*)‐**11** (95 % yield, 98 % *ee*).^[102]^ Taking into consideration the easiness of racemic synthesis, greener methods to easily access optically pure **11** may involve, in a future perspective, the use of Viedma ripening or temperature cycling,^[103]^ which have been only recently applied to the deracemization of axially chiral compounds,^[104]^ including a BINOL derivative.^[105]^

**Scheme 20:**
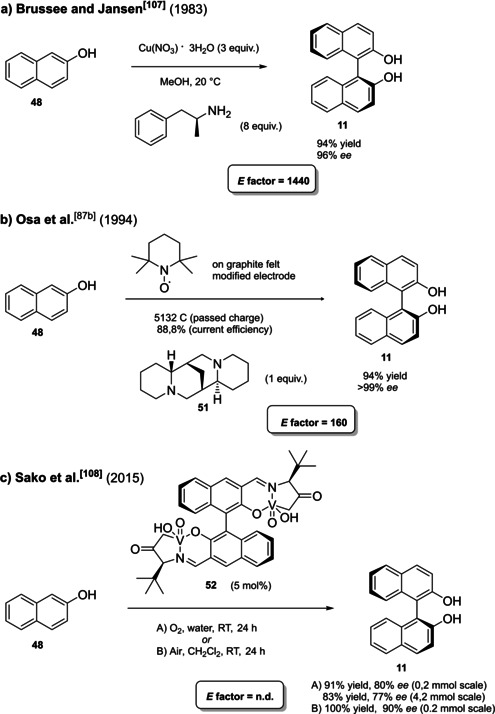
Successful protocols for (*S*)‐BINOL asymmetric synthesis.

For what concerns successful protocols of asymmetric synthesis, these are limited to the access to (*S*)‐**11** (Scheme 20).

Asymmetric synthesis of BINOL relies on oxidative coupling of β‐naphthol, whose proof of concept, although in low yield and optical purity, was given by Feringa and Wynberg in 1978.^[106]^ Five years later, Brussee and Jansen could afford (*S*)‐BINOL in 94 % yield and 96 % *ee* by oxidative coupling promoted by a chiral copper(II) complex with *D*‐amphetamine.^[107]^ This was the first high‐performing protocol to selectively obtain a single atropoisomer as useful product. Moreover, the reaction is carried out in methanol and mild reaction conditions, generating the catalyst in situ, albeit high amounts of expensive *D*‐amphetamine and tri‐hydrate cupric nitrate are employed. The *E* factor for the process is 1440, without taking into account recovering and recycling of the 80 % of the amine, as well as chemical waste deriving from its synthesis (Scheme 20a). Another interesting procedure is based on cyclic voltammetry electrooxidation on a TEMPO‐immobilized based graphite felt electrode (Scheme 20b). In this case, the almost perfect optical purity derives from the employment of natural‐occurring (−)‐sparteine **51**, which is recovered in 95 % yield and recycled.^[87b]^ In this case, the calculated *E* factor is 160, which could potentially be improved avoiding column chromatography, responsible for around 80 % of the total mass waste.^[108]^

**Scheme 21:**
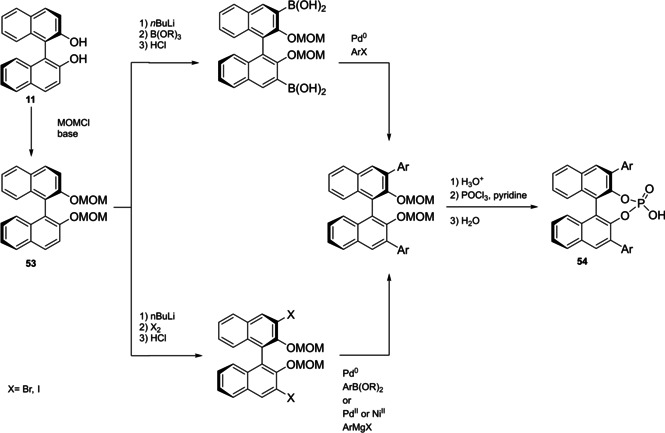
Standard protocols for the synthesis of BINOL‐derived phosphoric acids **54**.

Differently from the BINOL backbone, the synthesis of cyclic phosphoric acid diester derivatives is a quite standard synthetic route, involving: hydroxyl‐groups protection (e. g., like MOM‐ether **53**); lithiation followed by halogenation (bromination/iodination) or boronylation; Suzuki cross‐coupling with an aryl‐boronate or an aryl halide (bromide/iodide); ether hydrolysis; one‐pot phosphorylation and phosphoryl chloride hydrolysis (Scheme 21).^[109]^

**Scheme 22:**
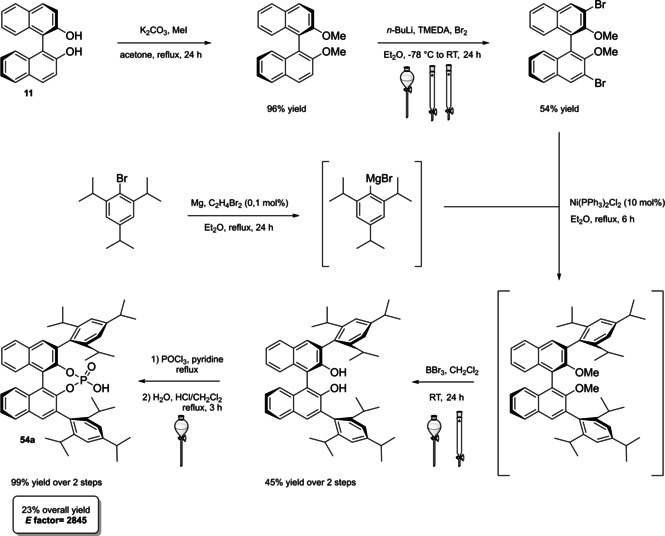
Standard protocol for the synthesis of **54 a**.^[78b]^

Calculation of the *E* factor in the case of BPAs is not possible in a general fashion, due to the strong dependence of the overall amount of waste on the selected protocol (*via* borylation or halogenation of **11** in 3,3’), the aryl group (preparation of the corresponding arylboronic ester or aryl halide), the source of Pd^0^ or Ni^II^, the selected protecting group and, obviously, reaction yield in each step. However, some criticisms can be highlighted with respect to the twelve principles. First of all, BINOL is not a natural feedstock (7^th^ principle), and the environmental impact of its synthesis has been evaluated by above mentioned *E* factor calculations. Second, no particular attention is devoted to the use of safer solvents and auxiliaries (5^th^ principle), as well as to reduce derivatives (8^th^ principle) and, thus, to atom economy (2^nd^ principle). Finally, critical or difficult reaction conditions are applied, implying higher risk (1^st^ and 12^th^ principle) and lower energy efficiency (6^th^ principle). To give a practical example, the *E* factor of TRIP‐PA (**54 a**),^[78]^ one of the most occurring BPAs organocatalysts,^[109]^ has been evaluated on the basis of a well‐established synthetic protocol (Scheme 22).^[78b]^

Starting from enantiopure **11**, introduction of methyl ether protecting groups on phenolic hydroxyl groups (against 2^nd^ and 8^th^ principle) occurs in nearly quantitative yield. In order to selectively brominate the 3,3′‐position, a classical ortho‐lithiation reaction, is performed under inert atmosphere at −78 °C, very far from energy efficiency conditions (6^th^ principle). After an extraction and two chromatographic purifications, Negishi cross coupling with a suitably generated Grignard reagent is performed, followed by protecting group removal, after which further extractive and chromatographic purification are required. Phosphorylation in pyridine with POCl_3_ finally allows to isolate the desired phosphoric acid **54 a** after a simple extraction, in 23 % overall yield with an *E* factor of 2845. The *E* factor of BINOL **11** is not taken into account in this calculation. Apart from the use of Grignard reagents and pyridine, whose safety issues have already been discussed above, it must be stressed the critical use of phosphorous oxychloride, which is a typical reagent for phosphorylation, but causes damage to organs upon prolonged exposure and is harmful if swallowed and fatal if inhaled.

**Scheme 23:**
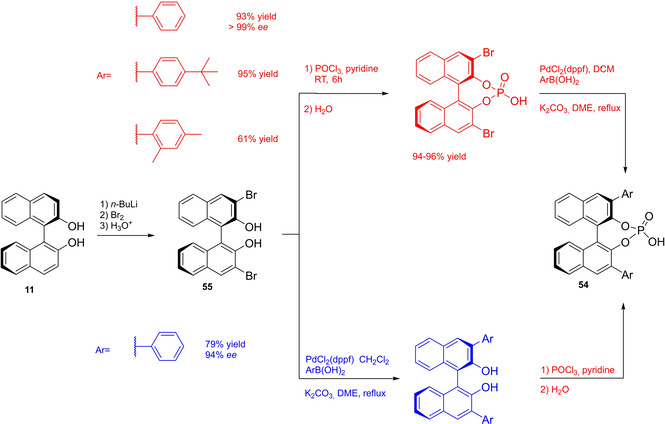
Protecting group‐free 3,3’‐functionalization of BPAs with aryl groups (dppf=1,1′‐Bis(diphenylphosphino)ferrocene, DME=1,2‐Dimethoxyethane).

Actually, it has been demonstrated that the use of protecting groups is dispensable, and that 3,3′‐substituted BPAs can be prepared either by the bromination–Suzuki cross‐coupling–phosphorylation or by the bromination–phosphorylation–Suzuki coupling sequence.^[110]^ In this way, starting from 3,3′‐dibromo/diiodo BINOL **55**, the number of steps could potentially be reduced to four. The procedure has however been applied just to a limited number of 3,3′‐hindered BPAs (Scheme 23).

**Scheme 24:**
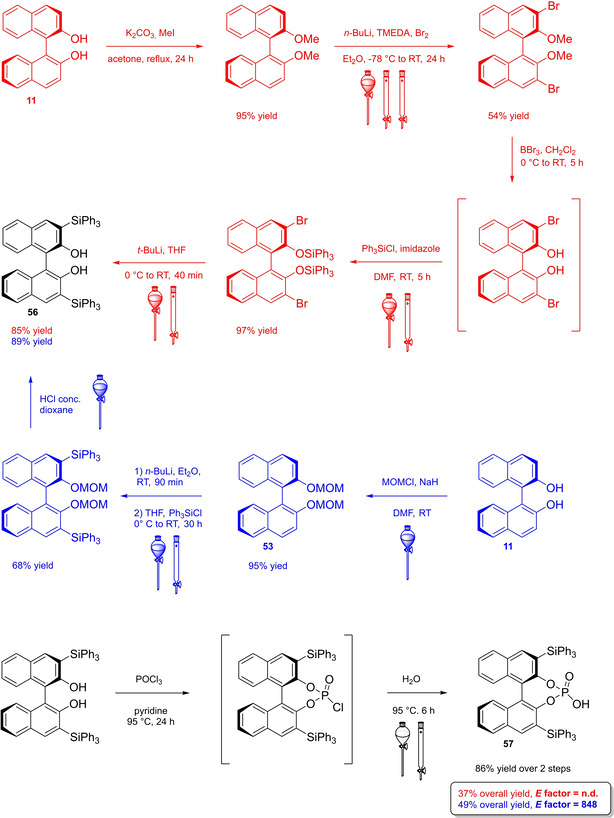
Alternative synthetic routes to **57** are depicted, respectively, in red and in blue. Common steps are depicted in black (TMEDA=N,N,N′,N′‐Tetramethylethylenediamine).

In 2018, Feringa and co‐workers proposed Pd‐promoted cross coupling to functionalize **11** with 3,3′‐substituents characterized by a lower *E* factor.^[111]^ Nevertheless, the mandatory employment of highly pyrophoric *t*‐BuLi strongly limits its safeness (1^st^ and 3^rd^ principles) and scalability. Another notable example of efficient 3,3′‐hindering substituents for BINOL is represented by bulky silyl groups. The very first route to *o*‐silyl derivatives of BINOL has been developed by Yamamoto in 1988 and relies on rearrangement of *o*‐bromo phenolic silyl ether functions to *o*‐silyl phenols.^[112]^ The resulting 3,3′‐bis‐silylated BINOLs, apart from being good chiral ligands, can be considered as well suitable backbones for new chiral BPAs, like 3,3′‐triphenylsilyl‐2,2′‐binaphthyl phosphoric acid (**57**) (TIPSY‐PA), reported for the first time by the MacMillan group.^[113]^ A more convenient route to 3,3’‐silylated BINOL derivatives was however exploited by MacMillan and co‐workers, including direct lithiation of BINOL‐MOM ether **53**.^[114]^ Several silylated BPAs can be obtained. As reported in Scheme 24, two possible routes to silylated BPAs are proposed, TIPSY **57** was chosen as model compound.

Both the routes include critical operations, with respect to the principles of green chemistry, the blue one presenting a reduced number of synthetic steps (5 against 7) and chromatographic purifications (3 against 4) together with higher overall yield (49 % against 37 %). Moreover, the use of highly pyrophoric *t*‐butyllithium is avoided in favor of handier *n*‐butyllithium, which is nevertheless a non‐green reactant too (3^rd^ and 4^th^ principles). Consequently, Yamamoto's route to TIPSY diol **56** (in red) was not considered in the calculation of the *E* factor for compound **57**, which is 848 according to MacMillan's route (in blue). To summarize, the access to 3,3’‐silylated BPAs can be slightly more atom‐economic and less waste‐producing than 3,3’‐aryl BPAs.

**Scheme 25:**
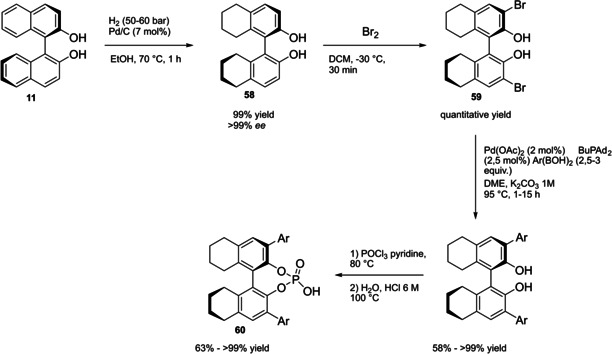
Access to H_8_‐BINOL‐phosphoric acids **60**.

Finally, modified BINOL‐scaffolds have been synthesized in order to tune the electronic effects and increase the steric bulkiness of BPAs, one of the most important being H_8_‐BINOL **58**, namely 5,5′,6,6′,7,7′,8,8′‐octahydro‐2,2′‐binaphthol. This scaffold can be directly prepared by hydrogenation of **11** over a heterogeneous catalyst like Pd/C (Scheme 25).^[115]^ Finally, the phosphoric acid functionality is installed by treatment with POCl_3_. High‐yielding protocols for the bromination of this substrate give access to its 3,3’‐dibromo derivative **59** ^[116]^ and, also in this case, a protecting‐group free strategy for the Suzuki cross‐coupling on the unprotected diol **58**, more compliant to the 8^th^ principle, has been reported (Scheme 25).^[117]^

**Scheme 26:**
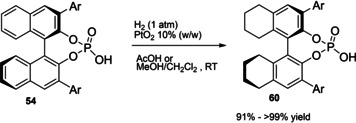
Direct reduction of BINOL‐PAs to H_8_‐BINOL‐Pas **60**.

The *E* factor of the overall process strongly depends on the aryl boronic acid and the yield of the last two steps. However, it is worth noticing that, despite an additional reduction step is required, with respect to the synthesis of simple BPAs, H_8_‐BINOL dibromo derivative **58** can be recovered in almost quantitative yield without the necessity of any *ortho*‐lithiation. Direct reduction of BINOL‐phosphoric acids to the corresponding H_8_‐BINOL‐phosphoric acids in high yield is also possible, although the reaction has been performed just on a sub‐mmol scale with a limited substrate scope (Scheme 26).^[118]^

#### 4.1.2 VANOL‐ and VAPOL‐based vaulted phosphoric acids (VPAs)

**Scheme 27:**
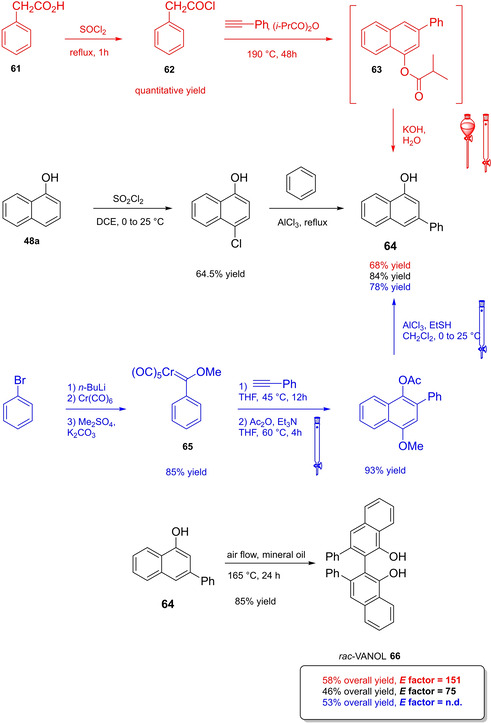
Alternative multi‐step syntheses of rac‐VANOL **66**.

3,3′‐Diphenyl‐2,2′‐binaphthalene‐1,1′‐diol (VANOL) (**66**) and 2,2′‐diphenyl‐3,3′‐biphenanthrene‐4,4′‐diol (VAPOL), commonly identified as vaulted biaryls, due to their complex three dimensional molecular structure, have been designed and synthesized for the first time by Wulff in 1993 to be employed as chiral ligands in Al‐catalyzed Diels‐Alder reactions.^[119]^ With the rise of asymmetric organocatalysis, VANOL and VAPOL have proven to be suitable scaffolds for hindered cyclic phosphates having narrow pockets for substrates, without the necessity of providing further functionalization by Suzuki, Negishi, or Kumada cross‐coupling. Currently, no asymmetric synthetic route exists to directly obtain optically pure vaulted biaryls, but several strategies have been proposed to access their racemate,^[120]^ which can be further resolved in the two atropoisomers or deracemized in a single enantiomeric form. All of these protocols have been optimized, so to provide standard ways to prepare these compounds in enantiopure form.^[121]^ Within the framework of VANOL and VAPOL synthetic routes, major issues concern the synthesis of the monomer building blocks. An open air, high temperature oxidative coupling of the monomer finally affords the corresponding racemic vaulted biaryl. Alternative routes to *rac*‐VANOL are outlined in Scheme 27. The first route, namely cycloaddiction/electrocyclization cascade (CAEC, depicted in red Scheme 27), starts from commercially available phenylacetyl chloride **62**, which can be obtained in quantitative yield by reacting phenylacetic acid **61** with thionyl chloride in CH_2_Cl_2_,^[122]^ with an optional catalytic amount of DMF.^[123]^ The following step relies on a [2+2] cycloaddition of the phenylketene generated by thermal decomposition of the acyl chloride and phenylacetylene. The resulting cyclic adduct **63** undergoes electrocyclic ring opening/ring closure, to be then trapped as an *O*‐acyl‐3‐phenyl‐α‐naphthol, which is finally hydrolyzed to give 3‐phenyl‐α‐naphthol **64**.^[120a]^ In the optimized version of the procedure, **64** can be obtained in 68 % yield after two crystallizations and a chromatographic column.^[121]^ The original alternative approach to compound **64** (depicted in blue in Scheme 27) includes synthesis and crystallization of a stable chromium carbene complex, which reacts with phenylacetylene in a benzannulation reaction, followed by acetylation to afford a stable naphthol derivative. Upon reaction with ethanethiol in the presence of AlCl_3_, methyl ether cleavage and reductive deacetylation give naphthol **64**.^[120]^ In terms of green chemistry, the latter alternative is even less preferable than the first one, since two chromatographic purifications are needed and critically toxic chromium‐based species are employed. However, the privileged protocol to naphthol **64** is the three‐step route via chlorination of cheap and commercially available α‐naphthol **48 a**, followed by AlCl_3_ in situ generation of cyclohexa‐2,5‐dienone and its rearrangement to naphthol **64** (depicted in black in Scheme 27).^[120]^ Employment of non‐green or even major issues VOCs (e. g., benzene) as the solvents is the only criticism; in return, despite a lower overall yield, the protocol is scalable on industrial scale, since no chromatographic purification is needed. Actually, chromatographic purification of residual mother liquor would increase the yield of the naphthol chlorination step from 64.5 % to 73 % and those of phenylation of 4‐chloro‐1‐naphthol from 84.1 % to 93.3 %, although increasing the total waste amount.^[121]^ Open‐air homocoupling of **64** finally leads to *rac*‐VANOL **66** in 85 % yield.^[120a]^

**Scheme 28:**
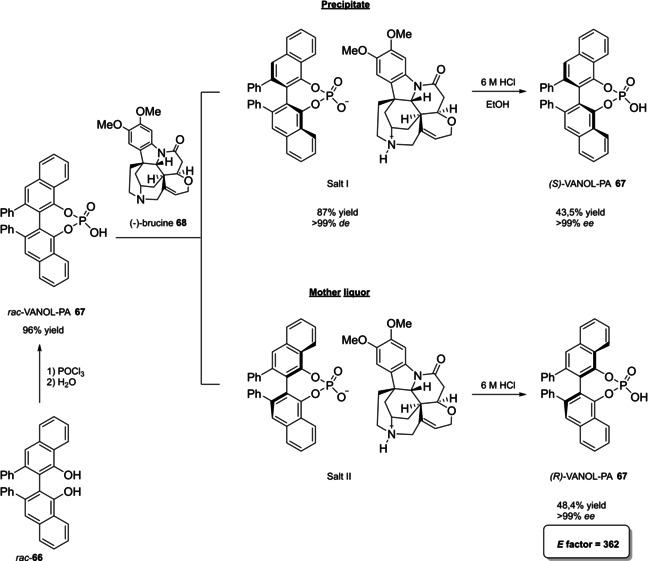
Optimized classical chemical resolution of *rac*‐VANOL **66**.

Classical phosphorylation into the corresponding atropoisomeric phosphoric acids and chemical resolution thereof is shown in Scheme 28.[^120a^, ^121^] Indeed, more waste would result from deprotection of VPAs to afford the two enantiopure VANOL atropoisomers, which formally represent the starting material for enantiopure VPAs.

Derivatization of *rac*‐**66** by phosphorylation and resolution of the atropoisomers with naturally occurring^[124]^ (−)‐brucine **68** potentially gives access to either the enantiopure acids by simple acidification of the salts with HCl (Scheme 28), or to the enantiopure forms of VANOL **66**, by treatment of the corresponding brucine phosphates with sodium bis(2‐methoxyethoxy)aluminum hydride (Red‐Al) in toluene at room temperature. Considering the route from α‐naphthol **48 a**, the *E* factor for enantiopure VANOL phosphoric acid **67** is 362, if both the enantiomers are assumed to be useful products. A more recent resolution strategy of VANOL describes the replacement of brucine with inexpensive *(*1*S*,2*S)*‐(+)‐cyclohexyldiamine, giving however poorer results in terms of yield and *ee*.^[124]^

**Scheme 29:**
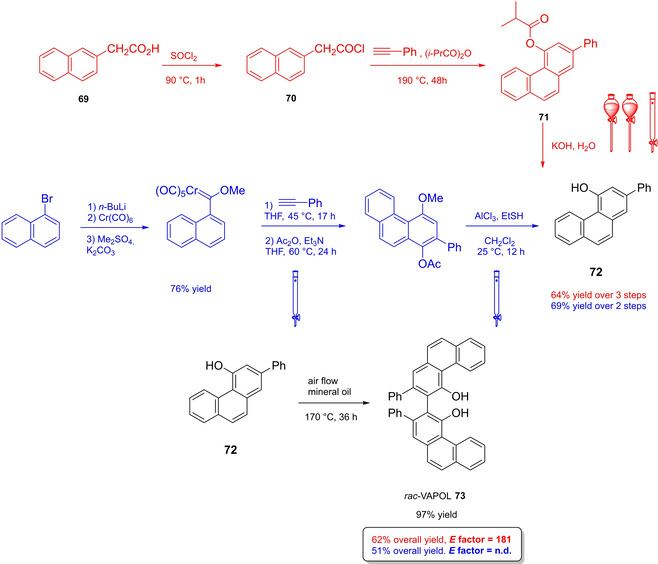
Alternative multi‐step syntheses of rac‐VAPOL **73**.

For what concerns the synthetic strategies towards 2‐phenyl‐4‐phenantrol **72**, key substrate in the synthesis of VAPOL, a single substantial difference exists with respect to those towards naphthol **64**. Indeed, while a low yielding chlorination of 4‐phenanthrol has been reported,^[125]^ the rearrangement of 1‐chloro‐4‐phenanthrol to **72** by refluxing in benzene in the presence of AlCl_3_ has not been investigated yet.[^121^, ^126^] Synthetic routes relying on CAEC or benzannulation of a chromium carbene complex are instead viable alternatives (Scheme 29).

**Scheme 30:**
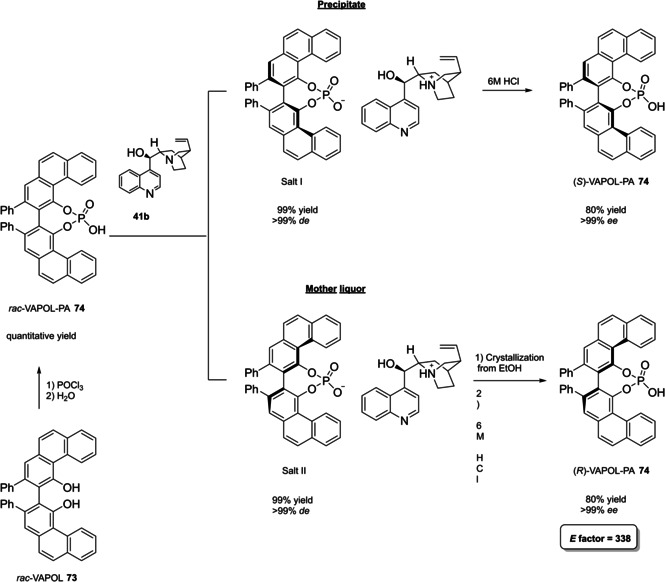
Optimized classical chemical resolution of rac‐VAPOL **73**.

CAEC from commercially available 2‐naphthylacetic acid (**69**) is a three‐step one‐pot procedure (depicted in red in Scheme 29), whose yield ranges between 57 % and 75 %, depending on the source of 2‐naphthylacetic acid, the reaction scale (14.2 g or 57 g) and, particularly, the purification method.^[121]^ Because of the absence of particularly hazardous or toxic reagents (excluding thionyl chloride), the limitation of the use of VOCs to the final purification step and the pot economy of the process, the CAEC approach can be considered the privileged one along the synthesis of VAPOL **73**. Taking into account a 57 g scale without any chromatographic purification, the best conditions allow to afford phenanthtrol **72** in 64 % yield. Remarkably, this route takes into account two extractive workups and a filtration on silica gel and Celite, which affect the *E* factor, despite the waste amount is lower than those of a true chromatography. Recovery of the unreacted starting material (**69** and SOCl_2_) was also considered in the calculation of the *E* factor, that is 181. The alternative route (depicted in blue in Scheme 29) makes indeed use of hazardous reaction conditions (lithiation of α‐bromonaphthalene at −78 °C), toxic chromium species, purification on stationary phases which are subsequently discharged and affords furthermore the final product in a generally lower yield.^[120a]^ Homocoupling of **72** to afford *rac*‐**73** was originally performed neat and open air between 190 and 210 °C to isolate the desired product in 80–89 % yield.^[120a]^ After optimization in mineral oil, the reaction temperature has been reduced to 170 °C and the isolated yield increased to 97 %.^[121]^ The resolution of the racemate resulting from phosphorylation in classical conditions follows an analogous approach to those above described for VANOL **67** (Scheme 30).

In this case, cinchonidine (**41 b**) is the selected chiral base to form diastereomeric salts with VAPOL phosphates and (*R*)‐ and (*S*)‐VAPOL are isolated in pleasantly higher yield than the enantiomers of VANOL‐PA **74** with equal enantiopurity and a final *E* factor of 338. This value accounts for both the atropoisomers of the phosphoric acid as useful products, taking into consideration the synthesis of VAPOL backbone from the simplest precursor **69**. In this case, it is remarkable the use of cheaper and more easily accessible **41 b** instead of **51**; moreover, the protocol completely avoids chromatography and is therefore suitable also for an industrial scale.

**Scheme 31:**
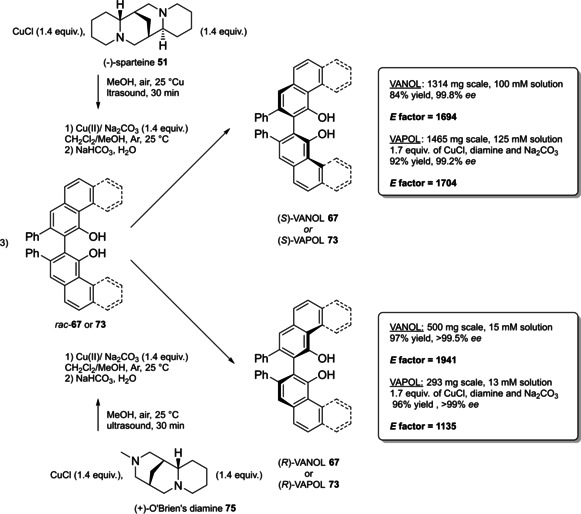
Optimized deracemization protocol for rac‐VANOL **67** and rac‐VAPOL **73**.

Overlooking chromatographic resolution on chiral stationary phases,^[127]^ the only currently available alternative strategy to afford VANOL and VAPOL in enantiopure form is deracemization.^[101]^ However, even if the optimized protocol is employed, the process is limited by a relatively small reaction scale, chromatographic purifications, high amount of solvents and use of non‐naturally occurring and highly expensive chiral bases (Scheme 31).^[101b]^

The *E* factors, in this case, reach considerably higher values, ranging from a minimum of 1135 for (*R*)*‐* **73** to a maximum of 1941 for (*R*)*‐* **67**. The deracemization is an example of dynamic thermodinamic resolution,^[128]^ based on *in situ* formation of a chiral Cu^II^ complex, for which two possible mechanistic pathways have been proposed. In terms of process economy, expensive synthetic chiral diamines have been replaced by Na_2_CO_3_ (scavenger for HCl) and NaHCO_3_ (quenching reagent) in those steps for which the chirality of the amine does not play an active role. Copper‐promoted thermodynamic deracemization leads to a single enantiomer of the vaulted biaryl ligand, but its effectiveness on a large scale has not been demonstrated yet, due to the necessity of deeper mechanistic investigations.^[101b]^ Therefore, taking also into consideration the high waste amount resulting from the sole deracemization of compounds **67** and **73**, the privileged way to access optically pure VPAs still relies on chemical resolution. Such phosphoric acids can be then convergently converted to the corresponding diols by treatment with Red‐Al without any loss of optical purity.^[128]^

Apart from VANOL **67** and VAPOL **73**, even more engineered biaryls and their derivatives have been synthesized: *iso*‐VANOL,^[129]^ 6,6’‐diphenyl VAPOL, 8,8’‐dimethyl VANOL and 8,8’‐diphenyl VANOL with their PAs,^[128]^ BANOL and its PA.^[130]^ However, excluding poor results in some screening,^[131]^ no significative applications of these VPAs (whenever the phosphorylation has been described) in asymmetric organocatalytic reactions has been reported so far, thus excluding them from the interest of the present review.

#### 4.1.3 SPINOL‐ and TMSIOL‐based spirocyclic phosphoric acids (SPAs)

**Scheme 32:**
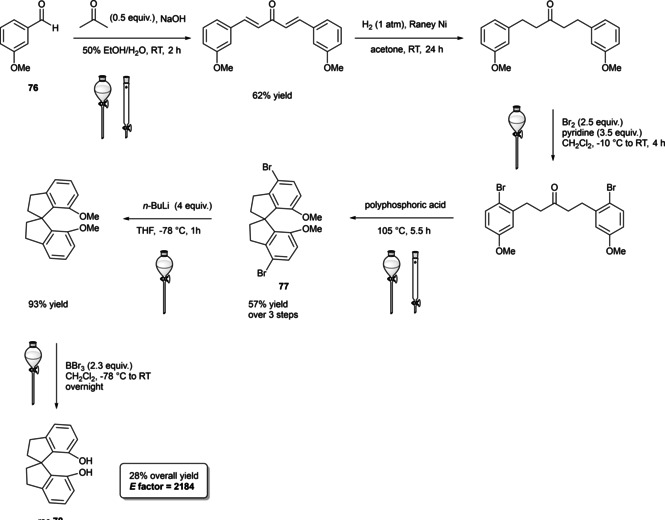
Original synthetic route towards *rac*‐SPINOL 76 by Birman et al.^[134]^

1,1′‐Spirobiindane‐7,7′‐diol (**78**) (SPINOL) has a special role as privileged chiral ligands.^[132]^ In fact, although it is not a biaryl compound, it bears a chiral axis, passing through the quaternary carbon. Moreover, apart being C_2_ symmetric, its spyrocyclic structure guarantees more rigidity with respect to biaryls,^[133]^ and the inversion of the handiness is virtually impossible. This feature might be helpful in reducing the number of conformers of the species, thus being ideal in the design of chiral catalysts. **78** was first synthesized by Birman et al. in 1999 (Scheme 32),^[134]^ taking the moves from earlier works focused on the obtainment of spirotriptindanes^[135]^ or spirobiindanes.^[136]^

**Scheme 33:**
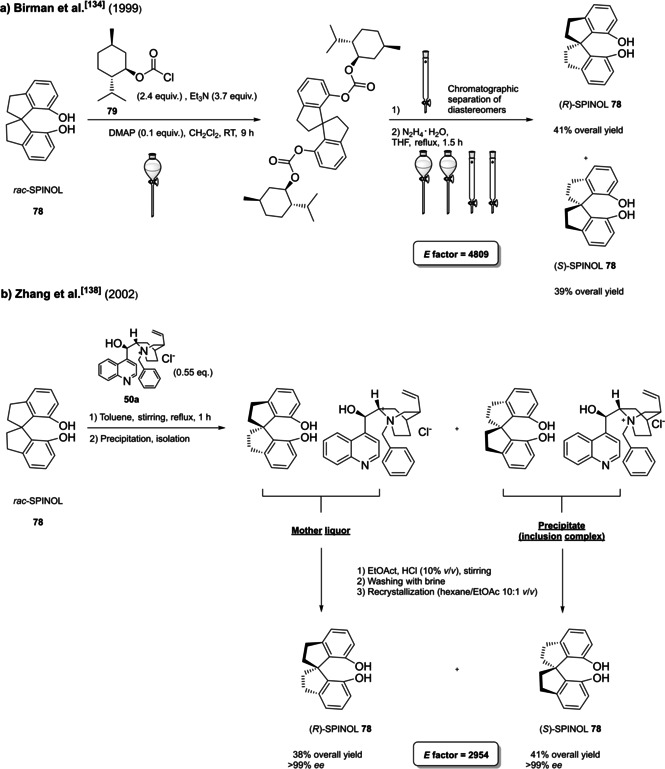
Alternative protocols for chemical resolution of **78**.

**Scheme 34:**
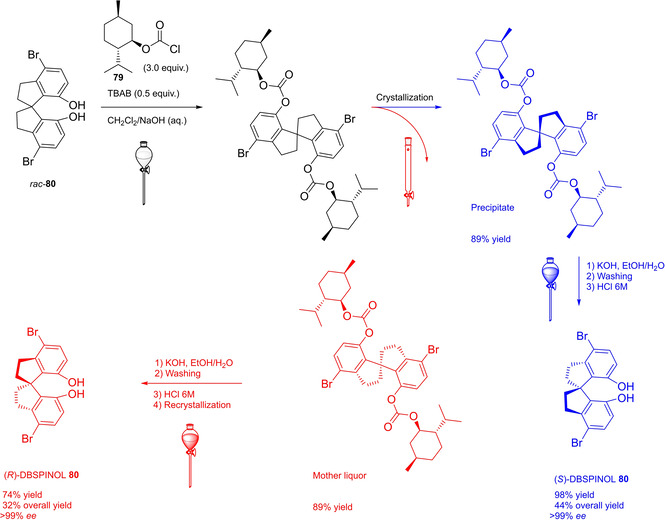
Chemical resolution of *rac*‐DBSPINOL by Zhang et al.^[139]^

A double aldol reaction of acetone and *m*‐methoxy‐benzaldehyde **76** in hydroalcoholic media and mild reaction conditions affords, despite in not excellent yield, a conjugated α,β‐ and α’,β’‐doubly unsaturated ketone. The latter product is then hydrogenated on Ni‐Raney catalyst and brominated in the *para* positions of the aromatic rings, with respect to methoxy groups, to afford the effective substrate of spirocyclization reaction. The latter is performed in polyphosphoric acid at 105 °C and gives compound **77**, the dimethyl ether of *rac*‐DBSPINOL **80** (namely 4,4′‐dibromo‐1,1’‐spirobiindane‐7,7′‐diol) in 57 % yield over three steps. The *p*‐bromination step is necessary in order to prevent the spirocyclization to occur *para* rather than *ortho*, and bromine atoms are subsequently removed by treatment with *n*‐BuLi at −78 °C, followed by methyl ethers cleavage with BBr_3_ at the same reaction temperature. Even in the case of SPINOL **78**, as well as for other privileged C_2_‐symmetric scaffolds, the synthetic route cannot be defined green, due to the employment of extreme reaction temperatures, volatile and chlorinated organic solvents, non‐easily handable reactants (e. g., *n*‐BuLi) and low overall yield. The *E* factor of 2184 is already high at this stage, but an even higher amount of waste is produced to isolate the enantiopure material, since no deracemization method has been developed. Basically, the obtainment of (*R*)*‐* and (*S*)‐SPINOL **78** still relies on chemical resolution. The only example of dynamic kinetic resolution of SPINOL, promoted by a N‐heterocyclic carbene, cannot be considered for the calculation of the *E* factor, affording the product only with 50 % *ee*.^[137]^ Two efficient methods of chemical resolution are currently being employed (Scheme 33). The first one was described in the original synthetic protocol by Birman et al., which includes derivatization of SPINOL as bis‐*L*‐menthylcarbonate, followed by flash column chromatography and menthyl carbonate cleavage, with almost complete recovery and recycling of *L*‐menthol (Scheme 33).^[134]^ The second one was, instead, reported in 2002 by Zhang et al., and it is based on inclusion crystallization with commercially available *N*‐benzylcinchonidinium chloride (**50 a**).^[138]^ The latter resolution method is preferable in terms of produced amount of waste. Moreover, in the Birman protocol, despite the synthesis of *L*‐menthylchloroformate (**79**) is not included in the calculations, the fact that hazardous phosgene is involved in the preparation of this derivatizing agent cannot be neglected. If this method of chemical resolution is selected, the *E* factor for the synthesis of optically pure SPINOL (**78**) is 4809. Chemical resolution by inclusion crystallization, on the other side, does not require derivatization, protecting group removal and column chromatography; however, the synthesis of the necessary amount of **50 a** shall be considered in the calculation of the final *E* factor, which is 2954. It is worth mentioning that, in 2004, Wan and co‐workers^[139]^ proposed a chemical resolution strategy for *rac*‐DBSPINOL (**80**), which is similar to those by Birman et al. for SPINOL, except for the additional employment of tetrabutylammonium bromide in the first step (Scheme 34). Access to **80** is pursued by exploiting Birman's route until dimethyl ether of DBSPINOL **77**, which is directly demethylated with BBr_3_ at −78 °C in 95 % yield, rather than being debrominated with *n*‐BuLi.^[139]^

**Scheme 35:**
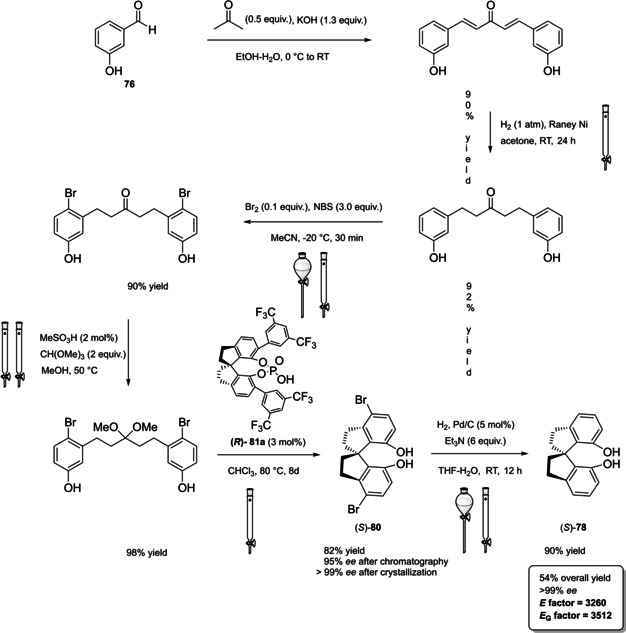
Asymmetric synthesis of SPINOL by Tan and co‐workers.^[140]^

The main advantage of this strategy resides in the possibility of avoiding column chromatography for the separation of (*R*)‐ and (*S*)‐bis‐*L*‐menthylcarbonates of DBSPINOL **80**, in favor of a simple crystallization followed by appropriate workup of the two phases. However, issues concerning the synthesis and the employment of derivatizing agent **79** represent a limitation also for this procedure. For this reason, no significative advantage results in terms of waste reduction with respect to inclusion crystallization. More recently, the Tan group developed the first asymmetric synthesis of SPINOL and SPINOL‐derivatives (Scheme 35).^[140]^

The Tan protocol involves a Brønsted acid‐promoted organocatalytic spirocyclization, with 3 mol % of a chiral SPINOL‐derived SPA **81** replacing the polyphosphoric acid. Performing the *E* factor calculation in the classical fashion, a value of 3260 results. However, the considerable *E* factor value of the phosphoric acid catalyst (see Scheme 37 makes necessary to include the catalyst waste mass in the calculation of a global *E* factor (*E*_G_ factor), whose value of 3512 is 7,7 % higher than the classical *E* factor (the last value does not include SPINOL **78** synthesis). This issue inspires the critical aim of this review, and it will be further examined in the last chapter, with some other selected examples.

**Scheme 36:**
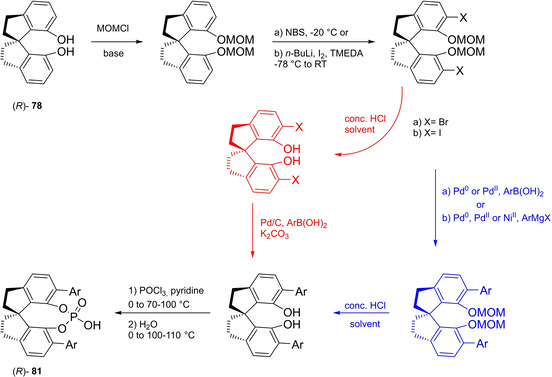
Standard protocols for the synthesis of SPINOL‐derived phosphoric acids **81**. Alternative steps are highlighted in different colors.

Despite not boosting the general greenness of the process, the work of Tan's group^[140]^ offers different advantages with respect to classic synthesis/resolution route to SPINOL **78**. First of all, the synthesis of the starting material appears to have been improved by the authors, who decided to change the substrate (*m*‐hydroxybenzaldehyde instead of *m*‐methoxybenzaldehyde) and the bromination procedure employing NBS and sub‐stoichiometric bromine in acetonitrile at −20 °C. These expedients allow to increase the yield of each single step to over 90 % and to avoid the final demethylation at −78 °C (cryogenic conditions against the energy efficiency pursued by the 6^th^ principle). DBSPINOL **80** is finally debrominated through hydrogenation catalyzed by palladium on activated charcoal, with a 54 % overall yield of the final enantiopure product. Notably, the (*R*)‐SPA **81 a** affords (*S*)‐SPINOL **78** and vice versa, but no substantial difference in the cost or environmental impact follows from this issue. With the enantiopure backbone in hands, preparation of phosphoric acid organocatalysts **81** follows a quite general route, similar to those of BPAs (Scheme 36).^[141]^

**Scheme 37:**
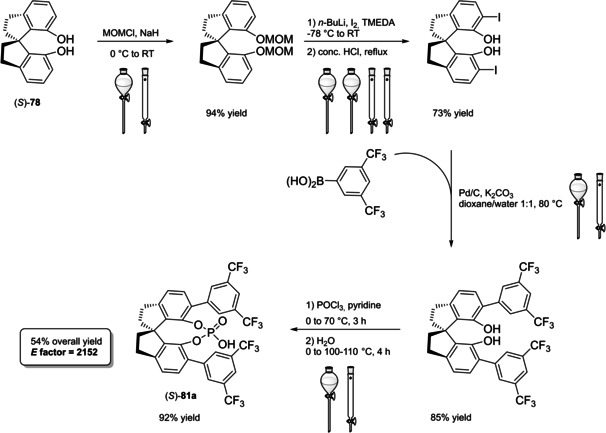
Xu et al. protocol for the synthesis of **81 a**.

After protection of the free hydroxyl groups as MOM‐ethers, halogenation in the positions 6 and 6′ of **78** provides a suitable scaffold for Suzuki or Kumada coupling. Bromination does not require to employ molecular bromine, replaced by NBS;^[142]^ while iodination shall be performed in harsher reaction conditions (−78 °C rather than −20 °C), since reaction with electrophilic molecular iodine priorly includes deprotonation of the aromatic rings with *n*‐BuLi.^[143]^ In both cases, reaction conditions do not fulfill the twelve principles of green chemistry, despite a temperature of −20 °C is certainly more manageable and the reaction can be performed by the use of a simple eutectic mixture of ice/sodium chloride in a thermostat dewar. The employment of palladium‐ or nickel‐based catalysts for Suzuki or Kumada coupling is instead itself respectful of the 9^th^ principle. After MOM‐ethers cleavage, standard phosphorylation/hydrolysis in dry pyridine, which in the case of SPINOL‐derived backbones is performed at temperatures between 70 and 110 °C, allows to afford the desired SPA **81**. Alternatively, MOM‐ether cleavage can be performed before the metal‐catalyzed cross‐coupling. Different SPAs **81** have been prepared with the aim of promoting organocatalytic reactions since 2010, when two approaches to their synthesis by Xu et al.^[144]^ and Čorić et al.,^[145]^ respectively, simultaneously appeared in the literature. In particular STRIP (**81 b**),^[145]^ an SPA analogue of **54 a**, has been recognized as the most used catalyst in the literature. However, since the majority of the route would be a reiteration of those to **54 a**, herein the complementary approach to the one of Xu et al.′s SPAs, namely 6,6′‐(3,5‐trifluoromethylphenyl)‐SPINOL‐PA (**81 a**), is described in detail (Scheme 37).

**Scheme 38:**
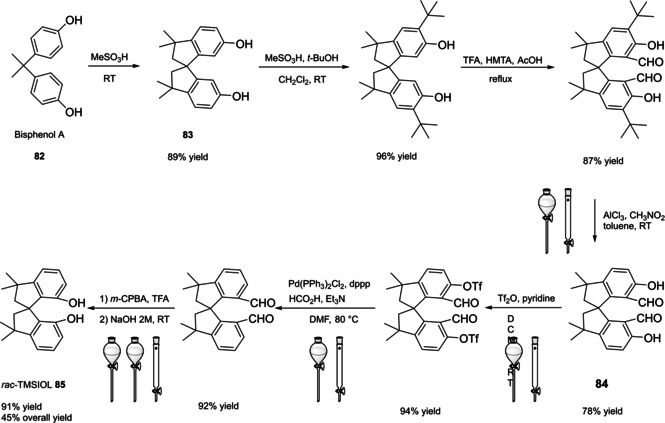
Protocol for the synthesis of **85**. (Dppp=Bis(diphenylphosphino)propane, m‐CPBA=meta‐chloroperoxybenzoic acid).

Free hydroxyl groups are protected with MOM chloride, and the resulting bis‐MOM‐SPINOL is iodinated in the positions 6 and 6’, following the classical procedure of lithiation/electrophilic addition of I_2_ at −78 °C. Bis‐MOM ether is hydrolyzed in concentrated hydrochloric acid, then Suzuki coupling is efficiently performed using catalytic palladium on charcoal. The preparation of arylboronic acid and its precursor 3,5‐bis(trifluoromethyl)benzene should be considered in the calculation of the *E* factor. However, since it is not reported by the authors, it will be omitted for simplicity. After the usual phosphorylation of the diol, followed by hydrolytic workup, the *E* factor of the final SPINOL phosphoric acid reaches a value of 2152. This particular phosphoric acid has been also prepared in a slightly different fashion,^[133a]^ although isolated in considerably lower yield. SPINOL‐derived phosphoric acids represent almost the wholeness of SPAs, since spirocyclic diols similar to SPINOL have not been functionalized to phosphoric acids or their derivatives so far.^[146]^ However, in 2019, the Lin group has reported the synthesis of a novel SPINOL‐derived compound, named TMSIOL (**85**) (3,3,3′,3′‐tetramethyl‐1,1′‐spirobiindane‐7,7′‐diol or, alternatively, 3,3,3′,3′‐tetramethyl‐SPINOL),^[147]^ whose scaffold has been used to design the first “2^nd^ generation SPAs” to catalyze the asymmetric synthesis of axially chiral *N*‐arylindoles.^[148]^ Synthesis of *rac*‐TMSIOL **85** is presented in Scheme 38.

**Scheme 39:**
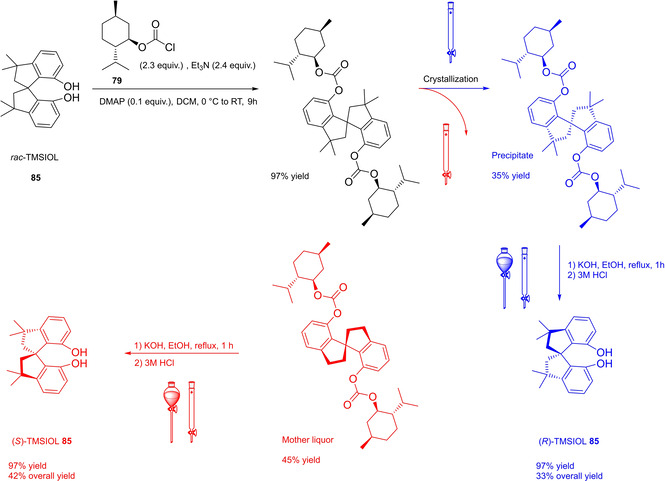
Protocol for the chemical resolution of TMSIOL.

Differently from those of SPINOL **78**, this synthetic route starts from bisphenol A **82**, which is a non naturally‐occurring substrate, but can be prepared through double condensation of acetone with two equivalents of phenol, without production of particular waste byproducts, except for water.^[149]^ Subsequent acid‐catalyzed rearrangement of bisphenol A on a multigram‐scale, following modified literature protocols,^[150]^ affords 6,6′‐TMSIOL (**83**) in 89 % yield. The latter product is then almost quantitatively functionalized with *t*‐butyl groups in the less hindered *ortho* positions (5 and 5’) by double Friedel–Crafts alkylation, in order to direct on 7 and 7’ positions the formyl groups insertion. Formylation is performed by Duff reaction with hexamethylenetetramine (HMTA) in 87 % yield. Retro Friedel–Crafts reaction with aluminum trichloride and nitromethane in toluene serves to remove both the *ortho t‐*butyl groups, yielding aldehyde **84** in 78 % yield. Free hydroxyl groups of aldehyde **84** are then esterified with triflic anhydride in 94 % yield and triflate groups are removed by palladium‐catalyzed reduction with formic acid in 92 % yield. Finally, a Baeyer–Villiger oxidation followed by hydrolytic workup gives *rac*‐TMSIOL (**85**) in 91 % yield, or rather 45 % overall yield over seven steps. Design and synthesis of **85** did not bring any particular benefit in terms of green economy since, despite a higher yielding protocol with respect to SPINOL **78**, the number of synthetic steps and, thus, of total waste amount is even higher. Furthermore, accessing higher molecular complexity implies also in this case to use protecting groups, toxic transition metals and major issues solvents, including pyridine and chlorinated VOCs. Chemical resolution of **85** relies again on derivatization as bis‐*L*‐menthylester, but, as in the case of DBSPINOL **77**,^[139]^ crystallization allows to avoid flash chromatography (Scheme 39).^[147]^

**Scheme 40:**
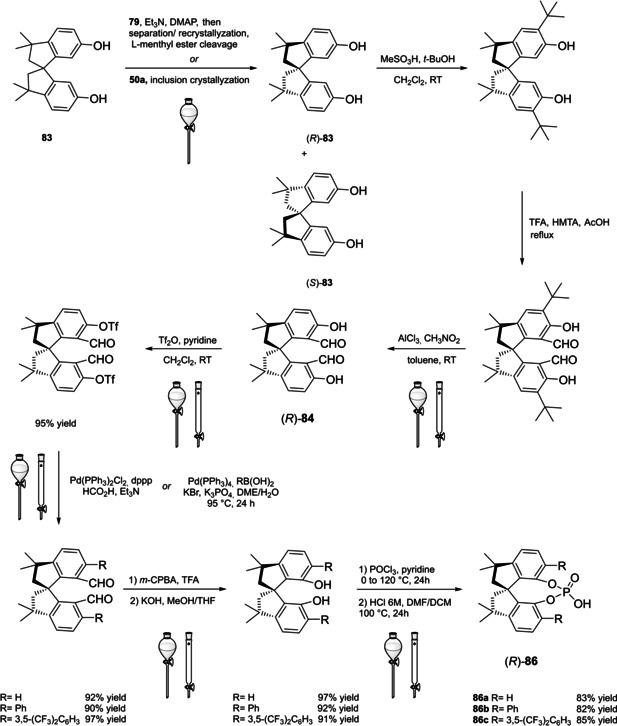
Synthetic route towards enantiopure 2^nd^ generation SPAs (^2^SPAs).

The synthesis of enantiopure 2^nd^‐generation SPA **86** currently requires to access enantiopure **85** in a different fashion,^[148]^ resolving intermediate **83** either through derivatization with *L*‐menthyl chloroformate **79** ^[151]^ or by inclusion crystallization with *N*‐benzylcinchonidinium chloride **50 a** (Scheme 40).^[152]^

The route is very similar to that depicted in Scheme 38, except for the fact that, despite the final product is a TMSIOL‐derived phosphoric acid **86**, enantiopure **85** is not isolated alongside its synthesis (except for ^2^SPA **86 a**). After the protection of free hydroxyl groups of aldehyde **84**, the final product is afforded by performing either the sequence reduction/phosphorylation/hydrolysis (^2^SPA **86 a**) or the sequence Suzuki–Miyaura coupling/phosphorylation/hydrolysis (**86 b** and **86 c**).^[148]^ Calculation of the *E* factor is not possible, since the authors do not give experimental details (including isolated yields) on the procedure for the synthesis of enantiopure **84**, referring to original protocols for the synthesis of HMSIOL derivatives (HMSIOL is the analogous derivative of bisphenol C). However, it is evident that the synthetic routes towards these novel phosphoric acids are neither inspired from sustainability, nor waste reduction aims. Even more engineered SPINOL‐derived phosphoric acids have been recently prepared with the purpose of designing novel MOFs, rather than of finding appropriate organocatalytic applications.^[153]^ For this reason, their synthesis will not be covered by this Review.

#### 4.1.4 Tartaric acid‐ and TADDOL‐based phosphoric acids (TPAs)

**Scheme 41:**
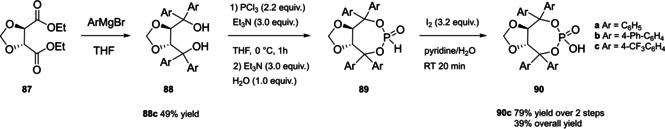
Synthetic route towards Akiyama et al.′s TPAs for asymmetric Mannich‐type reaction. Yields are given for product **90 c** and precursors thereof.

TADDOL is an acronym for α,α,α′,α′‐tetraaryl‐2,2‐disubstituted‐1,3‐dioxolane‐4,5‐dimethanol. Varying the substitution pattern of the dioxolane and the aryl groups, a wide variety of compounds fulfills this definition,^[155]^ thus defining a proper family of TADDOLs, whose precursor was first synthesized by Seebach et al. at the Eidgenössische Technische Hochschule (ETH) in Zürich in 1982 from the acetonide of dimethyl tartrate and phenylmagnesium bromide.^[154]^ Their easy accessibility from naturally‐occurring C_2_‐symmetric *D*‐ and *L*‐tartaric acid, makes them ideal to design ligands and catalysts in the respect of the 7^th^ principle of green chemistry. To the best of our knowledge, only four papers presenting TADDOL‐ or tartaric acid‐derived phosphoric acid organocatalysts (TPAs) have been reported so far.^[156]^ Other isolated examples of TPAs can be found in the literature, but their preparation and characterization has not been followed by any catalytic employment^[157]^ or, if yes, transition metals are part of the catalytic active system.^[158]^ Coherently, only the synthesis of the three efficient TPAs organocatalysts will be herein presented. It is however worth noticing that the massive employment of hindered BPAs, VPAs and SPAs in asymmetric organocatalysis, with respect to the scarce diffusion of TPAs, mainly deals with steric properties. Indeed, while the substitution pattern of the aryl groups helps in tuning the electronic properties of TPAs as well as of BPAs, VPAs and SPAs, TADDOL scaffold does not allow to design confined catalysts. The first examples of TPAs were reported in 2005 by Akiyama et al. (Scheme 41),^[156a]^ who is also a pioneer for design and use of BPAs.^[72]^

Akiyama et al. designed seven new TADDOL‐based TPAs, analyzing the effect of the aryl group and the acetal/ketal moiety on the catalyst backbone on the yield and the enantioselectivity of a known Mannich‐type reaction catalyzed by a BPA in his previous pioneering paper.^[72]^ Addition of an arylmagnesium bromide to an acetal or ketal of diethyl tartrate affords the desired TADDOL derivative. Since direct phosphorylation proved unsuccessful, a phosphinylation to phosphite **89** with PCl_3_ followed by oxidation with iodine and subsequent hydrolysis was selected as the synthetic strategy to isolate the final product. From the point of view of safeness (1^st^, 3^rd^ and 4^th^ principle), the employment of PCl_3_ presents similar drawbacks as POCl_3_ involving furthermore an additional oxidation step thus increasing the overall waste amount (against the 8^th^ principle). However, the authors did not report any experimental details, except for chemical yields in the case of the most effective TPA, in which the aryl group is *p*‐trifluoromethylphenyl. Nevertheless, comparing this synthetic route to those of a BPA, advantages in terms of green chemistry overcome the weak points represented by the low overall yield: both the enantiomers of the starting material are available from natural feedstocks, minor synthetic steps are required for their derivatization to the final product and no chlorinated solvents are involved in the procedures. The authors probably did not take into consideration the possibility of avoiding the use of VOCs, but since THF can be often replaced by green 2‐methyl THF or cyclopentyl methyl ether (CPME), the greenness of this route could be potentially improved. It was necessary to wait until 2016 to see the following application of TPAs in organocatalysis, this time by Widhalm and co‐workers, who designed a small library of 17 new TPAs to catalyse Mannich reactions with fluctuating results, although yielding the desired product in up to 96 % yield and 96 % *ee*.^[156b]^

**Scheme 42:**
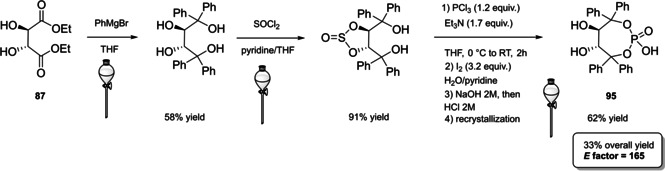
Synthetic route towards Hu′s TPA for asymmetric Biginelli reaction.

Once again, phosphorylation is performed following Akiyama protocol.^[156a]^ Alternatively, the authors perform a derivatization with 3‐hydroxypropionitrile immediately after the reaction with PCl_3_, followed by oxidation with hydrogen peroxide and cleavage with DBU. The yield of the final step is on average 10 % higher, but additional waste is produced because of derivatization. It is worth mentioning that, also in this case, the overall greenness of the procedure could be improved by finding greener alternatives to VOCs. One year later, Hu et al. designed novel tartaric acid‐derived phosphoric acids bearing free hydroxyl groups to promote a multicomponent asymmetric Biginelli reaction (Scheme 42).^[156c]^

Hu′s privileged catalyst **95** is not a TADDOL derivative, in fact the addition of phenylmagnesium bromide is directly performed on diethyl tartrate, then the hydroxyl groups of anti 1,1,4,4‐tetraphenylthreitol are protected by cyclosulfitation through addition of thionyl chloride. The sequence phosphinylation/oxidation/hydrolysis is even, in this case, a strategy to phosphorylate the diol. The last step is a non‐atom economic basic hydrolysis to remove the protecting sulfinyl group, followed by acidic neutralization to the desired TPA, in 33 % overall yield. The *E* factor of Hu′s catalyst is 165; no additional considerations are needed for the starting material, which is inexpensive and commercially available.

### 4.2 Phosphoric acid derivatives

The branch of Brønsted acid organocatalysis has been progressively researched for increasingly more acidic organic molecules in order to activate challenging and relatively inert substrates. Indeed, p*K*_a_ of such organocatalysts have been measured in different organic solvents,^[159]^ and their acidity has proven to be correlated to their activity, so that higher acidity of the catalyst leads to higher yields.^[75]^ In this scenario, derivatization of phosphoric acids to strongly acidic phosphoramides and phosphoric acid dimers (namely, imidodiphosphates IDP and imidophosphorimidates IDPi) always has an important impact on the eco‐sustainability of the overall synthetic route to the final catalyst. At this level of structure complexity, these catalysts possess high turnover numbers and non‐enantioselective background reactions are easily suppressed. However, if one has to pay attention to scalability of the process on an industrial scale or simply to green chemistry principles, the necessity of reducing the amount of waste imposes to carefully evaluate the best catalytic strategy.

**Scheme 43:**
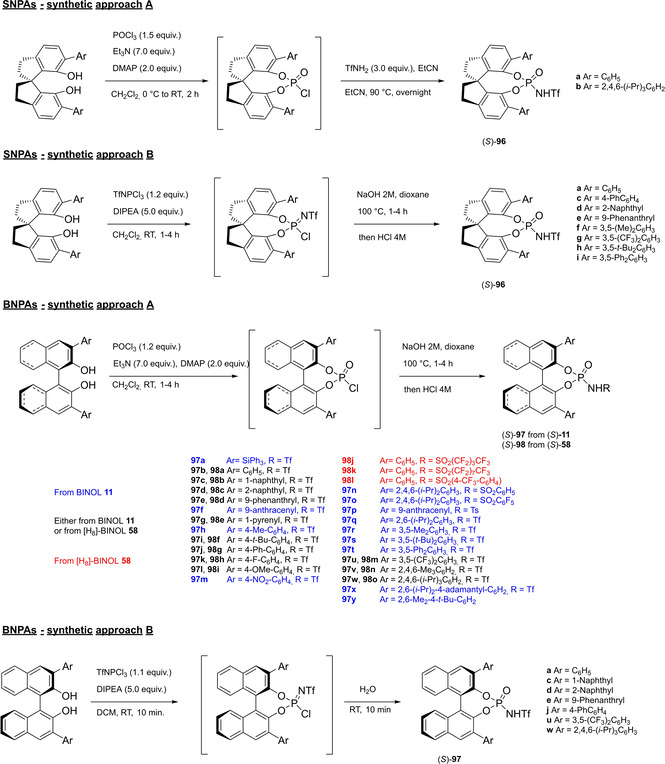
Synthetic approaches to BNPAs and SNPAs and products thereof.

Talking about phosphoramides, they were first presented by Nakashima and Yamamoto in 2006 as more acidic derivatives of phosphoric acids.^[160]^ In the most frequent case, they can be straightforwardly prepared by reacting phosphoric acid chloride with an appropriate sulfonamide (synthetic approach **A** Scheme 43), although more recent protocols employ phosphorimidoyl trichlorides as special phosphorylating reagents for BINOL/SPINOL and derivatives thereof (synthetic approach **B**).^[161]^ The great majority of phosphoramides has been obtained from a BINOL or a [H_8_]‐BINOL scaffold (BNPAs).^[162]^ A scarce number of phosphoramides has been synthesized from TPAs^[157b]^ or VPAs,[^128^, ^163^] without finding any successful application in asymmetric organocatalysis. On the contrary, STRIP‐*N*‐triflyl phosphoramide is the privileged organocatalyst for rearrangement of epoxides to aldehydes, [3+2]‐cycloaddition between hydrazones and alkenes, the one‐pot synthesis of tetrahydroquinolines with the simultaneous control on the formation of up to three stereocentres and the desymmetrization of oxetanes to tetrahydrothiophenes and tetrahydroselenophenes.^[164]^ Furthermore, seven novel spirocyclic phosphoramides (SNPAs) have been presented by Li et al. in 2019 for the sulfa‐Michael addition/enantioselective protonation of exocyclic enones.^[165]^ In this paper, phosphorylation approach with *N*‐triflylphosphorimidoyl trichloride is used for the first time to prepare a spirocyclic phosphoramide. The two synthetic approaches to axially chiral C_2_‐symmetric phosphoramides and relative products thereof are summarized in Scheme 43.

**Scheme 44:**
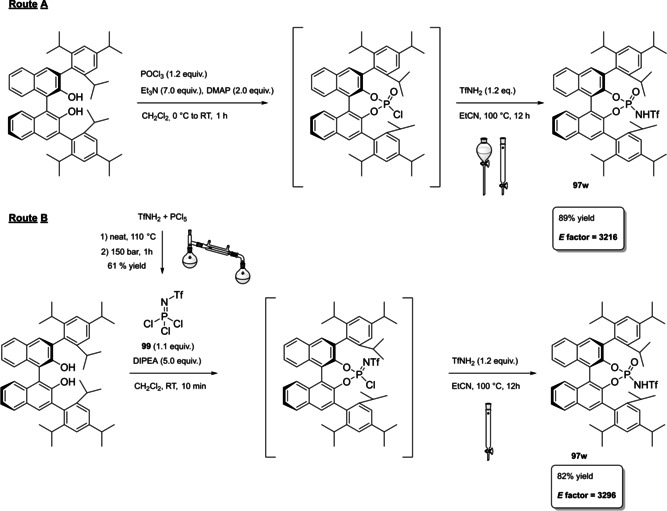
Comparison of synthetic routes **A** and **B** to TRIP‐BINOL phosphoramide **97 w**.

Comparing the two synthetic approaches for TRIP‐BINOL phosphoramide **97 w**, the *E* factor is 3216 with approach **A** and 3296 with approach **B**, the latter accounting for the synthesis of phosphorylating reagent *N*‐triflylphosphorimidoyl trichloride **99**. Synthesis of enantiopure **11** backbone is not included in these calculations and must be considered aside (Scheme 44).

Moving towards an increasing level of structure complexity and acidity of the catalysts, the synthesis of dual Brønsted acids, either diphosphoric acids^[166]^ or pyridylphosphoramides^[167]^ has been accomplished. However, a true milestone in organocatalysis has been achieved with the design of highly confined Brønsted acids, whose active site mimics the structure of an enzymatic pocket for substrates.^[168]^ Taking the moves from phosphorylphosphoramidates,^[169]^ imidodiphosphoric acids (IDPs) have first been proposed by the List group, these compounds display a rigid chiral pocket which is able to mimic the active site of enzymes even better with respect to phosphoric acids.[^170^, ^171^, ^172^] Only slightly later with respect to their very first appearance, Zhang and co‐workers also showed this new class of organocatalysts to promote an asymmetric three‐components Mannich reaction.[^174^, ^175^, ^176^] Because of the importance of confinement for stereoselectivity and the singularity, both for size and shape, of each catalyst pocket, no IDP has been instead prepared from VANOL **67**, SPINOL **78** and TADDOL, while the symmetric IDP of VAPOL **73** has been presented.^[178]^

**Scheme 45:**
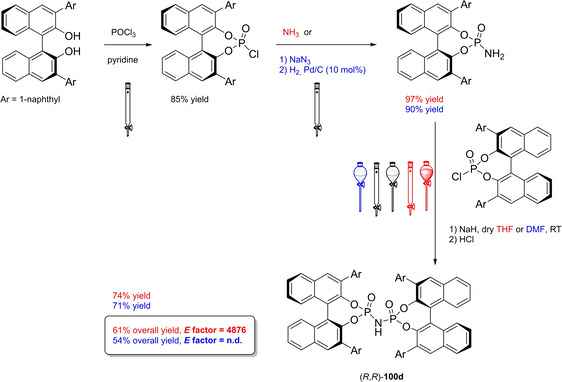
Synthetic routes towards IDP (*R,R*)‐**100 d**.

Since both the List and Zhang groups seem to have independently conceived the idea of a bis‐biaryl phosphoric acid dimer, two slightly different synthetic routes are available for the preparation of IDPs, both relying on the reaction of a biaryl phosphoramide with a phosphoryl chloride. Herein, the comparison of the two strategies is made by analyzing the preparation of IDP **100 d**, possessing bulky 1‐naphthyl 3,3′‐substituents. Indeed, this catalyst has either been used by Zhang and co‐workers for the asymmetric three‐components Mannich reaction^[174]^ and tested by List in the enantioselective acetalization of aromatic, benzylic and aliphatic aldehydes (Scheme 45).^[173]^

Excluding the common steps, the main differences are in the phosphoramide preparation from the corresponding phosphoryl chloride. Zhang approach includes homogeneous phase phosphorazide synthesis, followed by heterogeneous reduction with gaseous hydrogen on palladium/charcoal. Therefore, this route possesses no advantages with respect to phosphoramide synthesis with gaseous ammonia, in the absence of any transition metal species. Nevertheless, an environmentally impacting chromatographic purification is necessary in each case. Another minor difference can be found in the final step to **100 d**, when THF or DMF are alternatively employed: in both cases, the employment of greener reaction media would improve the fulfillment of the 5^th^ principle. Noteworthy, in the context of very similar yields for last step, employment of DMF requires an additional extraction to remove traces of this high‐boiling solvent (Scheme 45 in blue). Also, in the List route an additional sequence of chromatography/extraction shall be taken into account (Scheme 45 in red) increasing the overall waste amount and yielding a final *E* factor value of 4876.

**Scheme 46:**
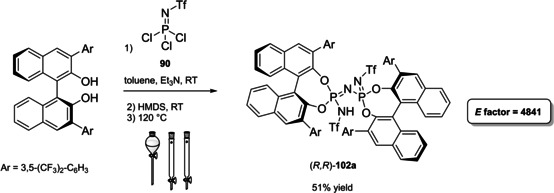
Synthetic route towards IDPi (*R,R*)‐**102 a**.

The last and most recent class of phosphoric acid dimers is represented by imidodiphosphorimidates (IDPi) **102**.^[177]^ No further insights will be given about these compounds, which have already been comprehensively reviewed,^[179]^ due to their important achievements in organocatalysis.[^180^, ^181^] For instance, IDPi were notably employed as Brønsted acidic catalysts to activate unbiased olefins by protonation^[182]^ and even promoted Diels‐Alder reaction in parts per million loading.^[183]^ Despite minimal possible modifications on the original protocol, the synthetic route is well‐established and relies on one‐pot phosphorylation of a proper 3,3′‐substituted derivative of **11** with a phosphorimidoyl trichloride, like compound **90**, and dimerization of the intermediate phosphorimidoyl chloride (which is not isolated) in the presence of HMDS. Other phosphorimidoyl trichlorides have been prepared and used by reacting PCl_5_ with an appropriate sulfonamide and purified as already discussed for compound **90**. In Scheme 46, the synthesis and the *E* factor of compound **102 a**, bearing two (3,5‐trifluoromethyl)phenyl groups as 3,3′‐susbtitutents, is depicted.

From an *E* factor of 4841,^[184]^ calculated from enantiopure **11** as the substrate, no particular disadvantages result with respect to IDPs. Phosphorimidoyl trichlorides cannot be defined green reagents but, at least, their preparation is industrially scalable since they can be purified by distillation without appreciably affecting the overall waste amount.

### 4.3 Chiral disulfonimides

**Figure 10:**
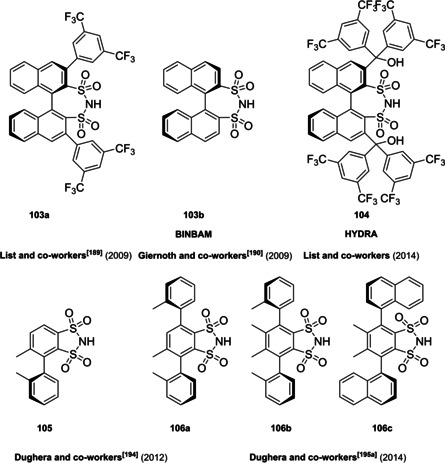
Representative chiral DSI from the two major classes: BINOL and non BINOL derived.^[191c]^

**Scheme 47:**
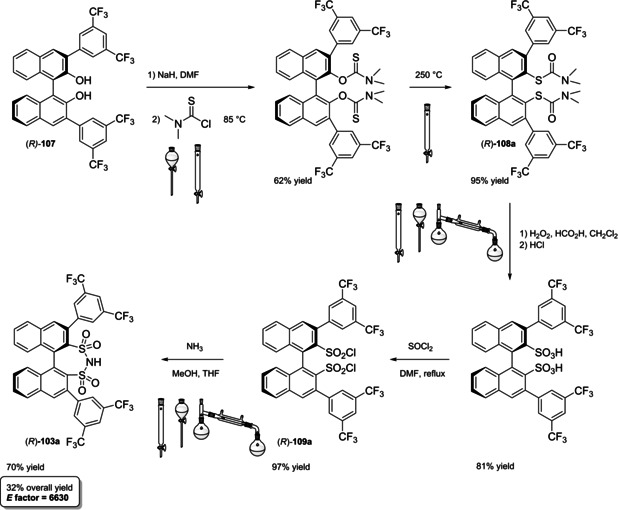
Original synthetic protocol for 3,3’‐diaryl‐BINOL DSI by List and co‐workers.^[189]^

Cyclic disulfonimide (DSI) does not only represent a sulfur‐based acidic functional group, but also the link between diverse phosphorous‐containing highly acidic species. Indeed, among the numerous sulfur acidic organocatalysts, the incorporation in a four to nine membered‐ring seems crucial to enhance the acidity of the sulfonamidic proton up to a strength comparable to those of mineral acids^[185]^ and generally overcame just by those of IDPis. The design of chiral DSIs takes the moves from achiral *ortho* benzene disulfonimide (OBS),^[186]^ whose synthesis was described since 1921,^[187]^ but that has been employed as a Brønsted acidic catalyst just in 2007, due to its excellent properties such as, apart from acidity, moisture insensitivity, safeness and high recyclability.^[188]^ Once again, the BINOL scaffold was exploited as a backbone for the first chiral DSIs, which were developed contemporarily and independently by List and co‐workers^[189]^ and Giernoth and co‐workers.^[190]^ Completely different and non‐confined C_2_‐symmetric chiral DSIs were later presented by Dughera and co‐workers,[^194^, ^195^] thus creating two classes of chiral DSIs (Figure 10), which have comprehensively been reviewed by List and co‐workers[^191a^, ^191c^] and Benda and France.^[191b]^ Access to the DSIs by Giernoth and List is possible by analogous functionalization routes. The original procedure starts from BINOL derivative **107**, whose hydroxyl groups are protected by reaction with thiocarbamoyl chloride after deprotonation with sodium hydride. Despite relatively high temperature, the reaction is not quantitative and extraction/chromatography sequence is necessary to isolate a bis(*O*‐arylthiocarbamate) intermediate. The following Newman–Kwart rearrangement is a valuable atom‐economic step due to the high reaction yield, in which the purification by column chromatography introduces a weak point in terms of waste amount. Oxidative cleavage of bis(*S*‐arylcarbamate) **108 a** with hydrogen peroxide is performed in the presence of CH_2_Cl_2_ and, after treatment with HCl, the bis‐sulfonic acid requires purification either by extraction, column chromatography and distillation, before being transformed in 84 % yield over two steps to the corresponding sulfonyl chloride **109 a** by refluxing in DMF with SOCl_2_. Finally, treatment with ammonia affords the final product **103 a** in 32 % overall yield, even in this case after extraction, chromatography and distillation (Scheme 47).

It is worth mentioning that Giernoth and co‐workers^[190]^ proposed an alternative approach also to this step. However, in contrast to the previous case, even for the choice of hazardous benzene as the solvent and for the use of gaseous ammonia source, the final reaction by List and co‐workers^[189]^ is preferable.

**Scheme 48:**
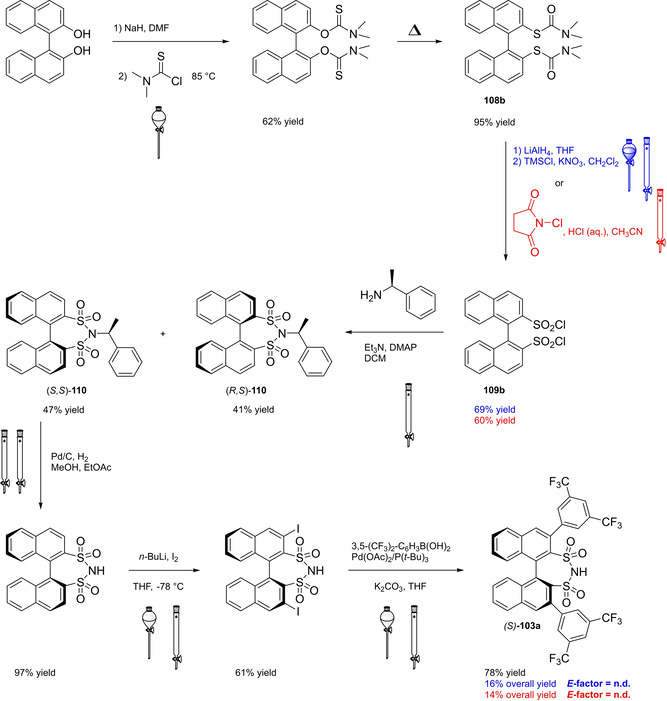
Late‐stage functionalization modified synthetic protocol for List DSI by Lee.^[193]^

**Scheme 49:**
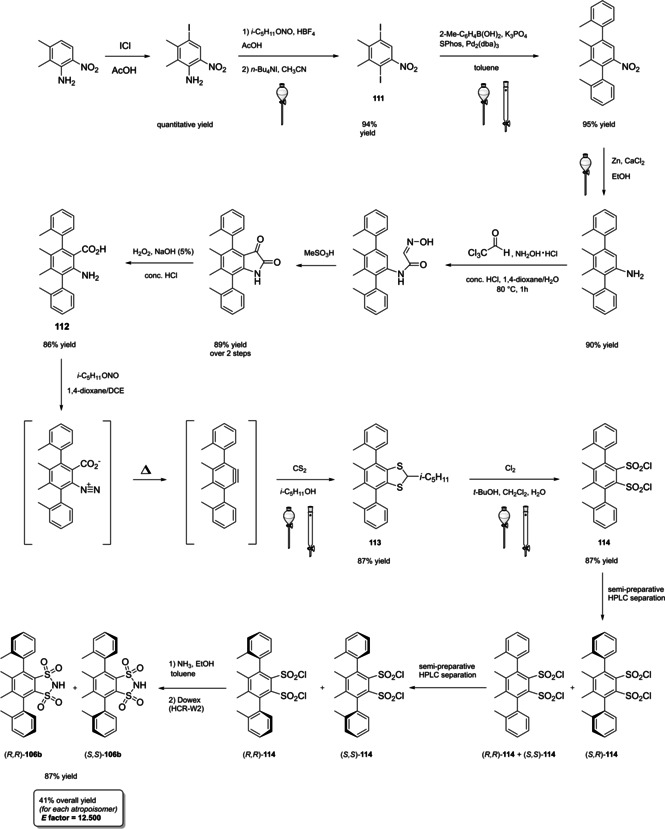
Synthetic route to bis‐biaryl cyclic DSI by Dughera (ICl=Iodine monochloride).^[195]^

The *E* factor of List's DSI following the described protocol is 6630. This value is not comprehensive of BINOL **11** preparation, but it includes the synthesis of compound **107** from **11**.^[192]^ Both the enantiomers of the catalyst are assumed to be useful products in the calculation. The choice of taking into consideration this preparation of **103 a** is due to the necessity to compare the original protocol described above^[189]^ with the modification proposed by Lee and co‐workers in 2010.^[193]^ Although retracing the same synthetic route as above, the authors employ racemic BINOL as the substrate. This approach alternatively transforms **108 b** in **109 b** using NCS in acetonitrile and HCl or by reduction with LiAlH_4_, (*S*)‐1‐phenethylamine is then selected as the resolution agent for the racemic bis‐sulfonyl chloride **109 b**. The resulting diastereomeric *N*‐benzyl DSI (*S,S*)‐**110** and (*R*,*S*)‐**110** are separated and debenzylated by hydrogenation with palladium on activated charcoal. Despite enantiopure BINBAM **103 b** is obtained in 97 % yield, two chromatographic purifications are necessary to obtain it in the necessary degree of purity, thus affecting the final waste amount. The final DSI is obtained in 14 % to 16 % overall yield, depending on the reaction conditions for the synthesis of **109 b**. Despite the two‐pot synthesis of **109 b** (Scheme 48) in blue allows to isolate the desired intermediate in appreciably higher yield, it presents no particular advantages. However, both of the possible routes do not suggest appreciable green improvements, since major issues solvents and non‐benign reactants and conditions are still present. Because of the lack of some necessary information (e. g., chromatographic data), the calculation of the *E* factor was not possible. For what concerns the design of the other major class of chiral DSI by Dughera and co‐workers, not dealing with BINOL **11**, a completely different strategy was followed, which can be described as a virtual 3,6‐installation of chiral axes on the achiral OBS backbone. The first OBS chiral derivative **105** dates back 2012^[194]^ and it is a biaryl DSI provided with *ortho* substituents, which prevents the molecule from racemization by free rotation around the stereogenic axis (Figure 10). Unfortunately, its employment within a three component Strecker reaction protocol resulted in poor enantioselectivity (32 % to 56 % *ee*). However, introduction of C_2_‐symmetry by installation of a second stereogenic axis, even in the absence of a confined structure (like in the case of 3,3’‐BINOL derivatives), afforded bis‐biaryl OBS derivatives, which notably gave excellent results as catalysts for atom‐economic multicomponent reactions, namely Strecker and Biginelli.^[195]^ The synthesis starts from 2,3‐dimethyl‐6‐nitroaniline, which is iodinated first on C_4_ with iodine chloride in acetic acid; then on the C1 atom, converting the amino group in the corresponding diazonium tetrafluoroborate salt with isopentyl nitrite in acetic acid and then performing a nucleophilic substitution with TBAI in acetonitrile. The *p*‐diiodonitroarene **111** is then obtained in high yield by simple reaction conditions and with a single extraction as the purification step. Furthermore, acetic acid, which is used as the solvent in two out of three steps, can be defined a good solvent in a sustainable chemistry perspective. The double Suzuki cross‐coupling to introduce *o*‐tolyl groups and, coherently, the two stereogenic axes is classically performed with a palladium catalyst, in the presence of SPhos as ligand, potassium orthophosphate as an additive and toluene as the solvent. Despite the high yield (95 %), chromatographic purification is necessary in order to filter off the exhausted catalytic system. Further reduction of nitroarene to aniline is carried out by reduction with metallic zinc and calcium chloride in ethanol in 90 % yield, followed by extractive workup. The employment of transition metal catalysts in classic volatile organic solvents may represent a weak point alongside the synthesis of a fully‐organic chiral catalyst; on the other side reactions proceed straightforwardly in mild reaction conditions. The following three steps aim to afford bis‐biaryl anthranilic acid (**112**) via isatin formation, following a classical Sandmeyer methodology, that cannot be properly defined a sustainable protocol, since reactions need to be carried out above room temperature and concentrated strong acids (HCl, MeSO_3_H) are employed. Nevertheless, chromatographic purifications are avoided and the following oxidative cleavage to product **112** can be conveniently performed in aqueous environment in satisfactory overall yield over three steps. Anthranilic acid is then transformed into the corresponding zwitterionic diazonium salt, which is subsequently converted into benzyne and reacted in situ with carbon sulfide in *iso*‐pentanol. After five steps, extractive workup and chromatographic purification are needed to isolate intermediate **113**, from which direct DSI precursor disulfonyl chloride **114** is finally afforded via reaction with gaseous chlorine in an aqueous mixture of *t*‐BuOH and CH_2_Cl_2_. Since the protocol does not enantioselectively introduce the two stereogenic axes on the molecule, two semi‐preparative HPLC run are necessary to separate the racemic mixture from the *meso*‐form and then the two enantiomers from the racemic mixture. This expedient has even worse impact on the *E* factor with respect to BINOL‐based catalysts, since the *meso*‐stereoisomer shall be inevitably discharged. Once the enantiomers of disulfonyl chloride **114** have been isolated, they can finally be converted into cyclic DSIs by reaction with gaseous ammonia in ethanol and toluene, followed by conversion into acidic form by ionic exchange with Dowex HCR‐V2 resin (Scheme 49). The *E* factor of compound **106 b** is 12.500, considering only the two enantiomers as the useful products.

In conclusion, despite the high stability, the safeness and the recyclability of the achiral precursor OBS have been extensively demonstrated, thus providing promising features for a “green” catalyst, both BINOL and non BINOL‐derived chiral DSI suffer from common disadvantages related to their synthetic routes. No attention is devoted to avoiding the use of unsafe reactants (e. g., carbon disulfide, gaseous chlorine, chloral), large amounts of common volatile solvents and chromatographic purifications.

In 2010, cyclic sulfurylimides, possessing a slightly different functional group with respect to DSI, have been presented by Berkessel et al. as a new and easier to synthesize class of Brønsted acid organocatalysts (JINGLE).^[196]^ Nevertheless, no enantioselective reactions promoted by this family of catalysts has been reported up to date and, for this reason, their synthesis will not be analyzed.

## 5 Hydrogen Bond Catalysts

### 5.1 Chiral thioureas and squaramides

**Figure 11:**
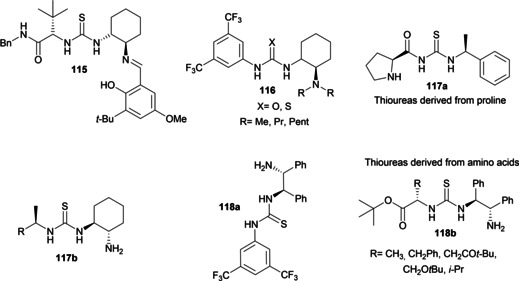
Selected examples of chiral thioureas based catalysts.

In 1998 Sigman and Jacobsen were the first authors to report the employment of a chiral thiourea as catalyst,^[198]^ a compound able to exert enantiocontrol through hydrogen‐bond interactions.^[198]^ Compound **115** paved the way to the development of a branch of organocatalysis based on non‐covalent interactions (Figure 11). A lot of work has been devoted to structure optimization and the introduction of the *N*‐trifluoromethylphenyl substituent allowed to tune the catalyst activity, solubility and rigidity, thus obtaining bifunctional compounds. As it can be seen from Figure 11 and Schemes 51–53, chiral thioureas/ureas catalysts are characterized by a central thiourea/urea functional group flanked on one side by an *N*‐trifluoromethylphenyl substituent or a chiral moiety derived from a protected amino acid or a chiral primary amine; the other side is derivatized with a chiral 1,2‐diamine. The catalyst is generally constructed reacting a preformed isothiocyanate, derived from an amino acid, 3,5‐bis(trifluoromethyl)phenyl amine or from a primary amine, with the chiral 1,2‐diamine that can be both a cyclic or acyclic compound.^[199]^ Moreover, the 1,2‐amine employed can be free or monoprotected allowing to obtain Jacobsen type thioureas **115** and **116** or bifunctional primary and secondary amine thioureas **117** and **118**.

**Scheme 50:**
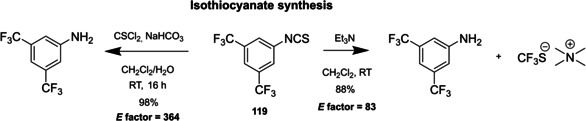
Different approaches for the synthesis of **119**.

The major concerns about thiourea synthesis arise from the preparation of the corresponding isothiocyanate which employing toxic and inflammable reagents go against the 3^rd^, 4^th^ and 12^th^ principles. Let us get down with isothiocyanate synthesis details. These compounds can be accessed reacting a primary amine with a high electrophilic source of C=S, such as thiophosgene (CSCl_2_), carbon disulfide (CS_2_) or dipyridylthionocarbonate and thiocarbonyldiimidazole. These reagents do not appear to be the best to meet the green chemistry principle. CSCl_2_ not only requires control of addition rate and temperature reacting exothermically, but it is also a highly toxic liquid.^[200]^ CS_2_ is flammable, volatile and needs stoichiometric co‐reagents or elevated reaction temperature.^[201]^ The last two points recall, also, the lack of atom economy and energy efficiency. An alternative to these reagents is tetramethylammonium trifluoromethanethiolate ((Me_4_N)SCF_3_) introduced by the group of Schoenebeck in 2017 (Scheme 50).^[202]^ This reagent also received the EROS best reagent award in 2020. (Me_4_N)SCF_3_ is, in fact, a solid and bench‐stable reagent, which allows the introduction of the isothiocyanate functionality with high functional group tolerance, high speed and efficiency. Moreover, the isothiocyanate can be isolated simply filtering out the salt byproducts. From a point of view of a greener protocol, CH_2_Cl_2_ could be furthermore substituted with not VOC solvents such as MTBE. Scheme 50 compares two different synthetic procedures which can be applied for the synthesis of 3,5‐bis(trifluoromethyl)phenyl isothiocyanate **119**. On the left side, the classical methodology based on thiophosgene is reported,^[203]^ while on the right side Schoenebeck protocol is presented.^[202]^ As it can be seen from Scheme 50, the latter avoiding the employment of toxic reagents and product purification allows to reduce the *E* factor to 83. On the contrary, in the presence of thiophosgene the methodology is not only unsafe but also characterized by a higher *E* factor of 364.

**Scheme 51:**
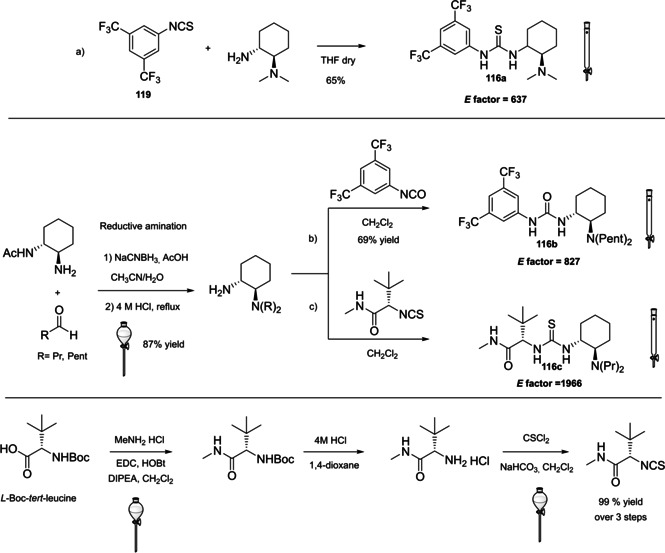
Reaction scheme for the synthesis of Jacobsen based‐thioureas (EDC= 1‐ethyl‐3‐(3‐dimethylaminopropyl)carbodiimide).

**Scheme 52:**
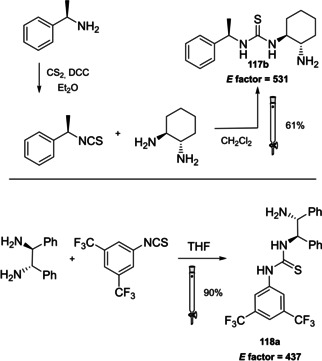
Synthetic pathways to bifunctional primary amine thioureas.

According to the complexity of the substituents on the thiourea moiety, isothiocyanate synthesis can be straightforward or lengthy. Generally, the employment of amino acids requires more steps because of protection and deprotection of sensitive functional groups with a general increase in the *E* factor and non‐compliance to the 2^nd^ and the 8^th^ principle (Scheme 51). As concern the 1,2‐diamine structures the most employed are: (1*S*)*‐trans*‐1,2‐diaminocyclohexane and (1*S*,2*S*)‐(−)‐1,2‐diamino‐1,2‐diphenylethane. As shown in Scheme 52, the production of bifunctional primary amine thioureas is quite simple, once the isothiocyanate has been prepared, it is directly reacted with the chiral 1,2‐diamine in CH_2_Cl_2_ or THF. The thiourea catalyst is obtained after purification on column chromatography with an *E* factor between 437 and 531, according to the product **117 b**/**118 a** yield.^[204]^ As shown in Scheme 51, if the amine functionality is derivatized, the *E* factor can rise, according to structure complexity, to 637 or up to 1966. In fact, the conversion of a primary amine in a tertiary amine functionality is realized by reductive amination of the corresponding mono‐acylated amine, which accounts for three additional synthetic steps. Moreover, the protection/deprotection strategy goes against the 2^nd^ and 8^th^ principles of green chemistry.^[205]^

**Figure 12:**
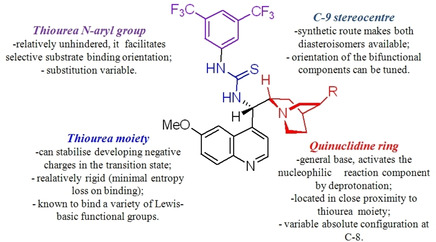
Thiourea‐modified Cinchona alkaloid catalysts: design elements.

The usage of Cinchona alkaloids as substrates on which introduce other functionalities, as shown in Scheme 17, opens up pathways to a variety of catalysts.^[206]^ Between them, the introduction of a thiourea or a squaramide group has several advantages. One of the most interesting features is their availability in two pseudo‐enantiomeric forms. Besides, *Cinchona* alkaloids are inexpensive starting materials obtainable from renewable feedstock with relatively rigid structures, in which Brønsted basic and hydrogen bond‐accepting functionality are located at stereogenic centers in close proximity to one another.[^197a^, ^207^] In this way, Cinchona alkaloid‐derived thioureas and squaramides can be considered bifunctional organocatalysts since the quinuclidinic ring of the alkaloid can act as a base allowing the activation of the nucleophile. On the other hand, the thiourea or squaramide group allows the activation of the electrophile and the stabilization of negative charges in the transition state by hydrogen bond formation (Figure 12).

**Scheme 53:**
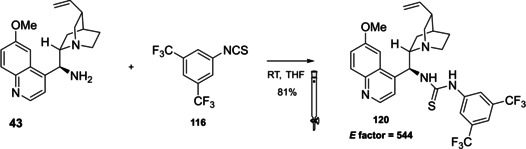
Reaction scheme for the preparation of thiourea **120**.

This compound can be obtained from 9‐amino(9‐deoxy)*epi*‐quinine (**43**) and 3,5‐bis(trifluoromethyl)phenyl isothiocyanate (**116**) in mild reaction conditions. If we consider that the starting materials for this reaction can be obtained from procedures characterized by low *E* factors (**43** *E* factor 150; isothiocyanate *E* factor 83), the only drawback is the purification of thiourea **120** by column chromatography, which implicates the increase of the *E* factor to 544 (Scheme 53).

**Scheme 54:**
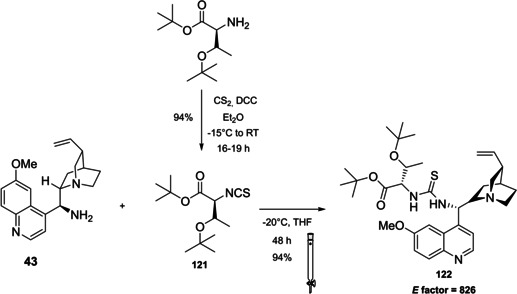
Multifunctional thiourea **122** derived from Cinchona alkaloid and amino acids. The *E* factor has been calculated for the catalysts derived from threonine.

Another class of multifunctional catalysts can be obtained flanking the key thiourea moiety by two chiral units, one derived from the *Cinchona* alkaloid the other from an amino acid. These compounds can be synthesized from the direct reaction between **43** or 9‐amino(9‐deoxy)*epi*‐cinchonidine with an isothiocyanate derived from a protected amino acid (Scheme 54).^[208]^

Apart for the lower reaction temperature necessary to avoid the epimerization of the chiral isothiocyanate, the approach pursued is the same of thiourea **120**, included the purification by column chromatography. An *E* factor of 826 can be justified considering that the isothiocyanate **121** is obtained reacting the protected amino acid with carbon disulfide and a stoichiometric amount of DCC, thus the isothiocyanate itself, prior to use, requires purification by filtration on silica gel, increasing the amount of waste produced.

**Scheme 55:**
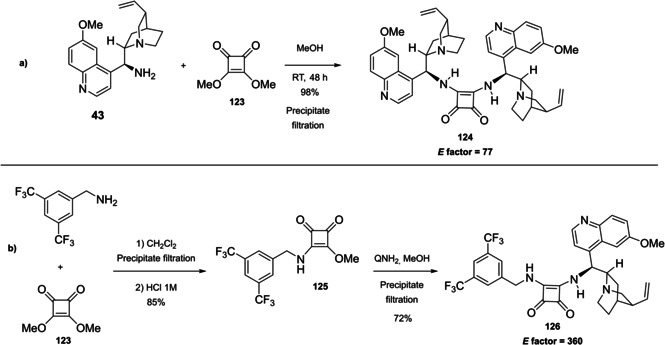
Reaction schemes for the preparation of squaramides **124** and **126** derived from Cinchona alkaloids.

The bifunctional nature of thiourea‐based organocatalysts can represent a limitation for the catalytic activity itself, since H‐bonded aggregates can be formed in dependence of concentration and temperature.^[209]^ In order to prevent self‐aggregation of the catalyst, Song and co‐workers introduced squaramide based dimeric Cinchona alkaloids **124**.^[210a]^ These compounds can be easily accessed by the one‐step reaction of dimethyl squarate **123** with one of the 9‐amino‐(9‐deoxy)‐*epi*‐Cinchona alkaloids (Scheme 55a). Being known to produce severe contact dermatitis,^[211]^ precautions should be taken when employing dimethyl squarate. Nevertheless, the procedure reported is characterized by high efficiency (98 % yield), atom economy, energy efficiency (all reactions are run at room temperature), and the production of waste is limited since the product can be filtered out from the reaction mixture. These features contribute to a very low *E* factor of 77.

In addition to dimeric catalysts, the squaramide moiety can incorporate two different amine functionalities. As shown in Scheme 55b, these dual hydrogen‐bond catalysts can be obtained in a two steps reaction. In the first step, the less hindered amine is added to dimethyl squarate **123** in CH_2_Cl_2_, the mono‐substituted product **125** is precipitated and, without further purification, it is subjected to a second substitution reaction in the presence of 9‐amino‐(9‐deoxy)‐*epi*‐quinine/cinchonidine. Even in this case, the disubstituted squaramide **126** precipitates out of solution. With respect to the dimeric squaramide synthesis, the production of disubstituted squaramides is characterized by a higher *E* factor (77 vs. 360), but for this increment one should take into consideration the presence of an additional reaction step and purification.^[212]^

**Scheme 56:**
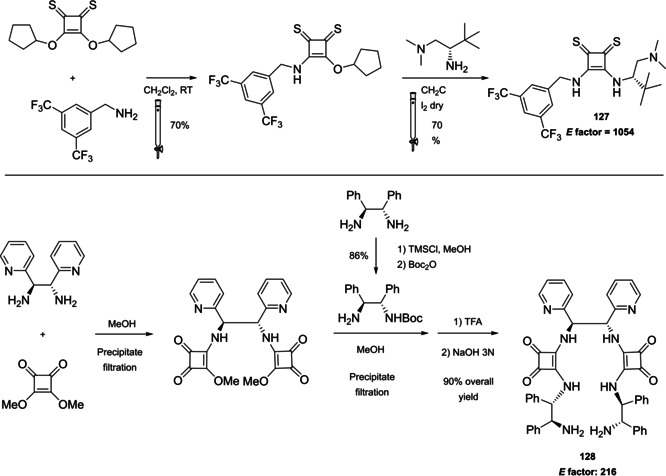
Comparison between different approaches to obtain bifunctional squaramides.

Among bifunctional catalysts, squaramides are characterized by a dual hydrogen‐bond donor and acceptor ability, a more rigid structure and lower spacing between the N−H groups than thioureas which, forcing the amine and carbonyl groups to be coplanar, thus limiting conformational mobility of the catalyst. In general, squaramide structures are more easily accessible than thioureas; in fact, the primary amine can be reacted directly with dimethyl squarate. According to the ratio amine/dimethyl squarate, it is possible to access dimeric or disubstituted derivatives. As seen for squaramides derived from Chincona alkaloids, in general, *E* factors are higher for the disubstituted catalyst, for which additional synthetic steps are necessary to insert selectively two different amine functionalities, one of them being chiral. Independently from the structure complexity, the squaramide can be easily purified through precipitation employing MeOH as solvent (*E* factor 216; Scheme 56).^[213]^ As shown in Scheme 56 for the thiosquaramide **127**, if CH_2_Cl_2_ is employed instead of MeOH, the product precipitation does not occur and a column chromatography is necessary, causing an increase in the *E* factor (1054).^[214]^

## 6 Lewis Acid Catalysts

Since the classification of the organocatalysts is made on the basis of their mechanism of substrate activation, rather than on their structure, many categories of organocatalysts can be differently classified as Brønsted or Lewis bases or acids depending on the context. For what concerns Lewis acids, this is for instance the case of phosphoric acids, whose mechanism may involve coordination of reactants by hydrogen bond, rather than a real proton transfer, meanwhile the oxygen of the double bond P=O, if involved, plays the role of a Lewis base.^[109]^ Furthermore, other versatile compounds here classified as Brønsted acids may act like Lewis acid pre‐catalysts,[^177^, ^215^] in the presence of silylated reactants: this is the case of IDPis and DSIs.[^10^, ^179^] Therefore, the synthesis of these compounds will not be discussed again, but all the comments made in the previous chapter remain valid.

## 7 Chiral Phase Transfer Catalysts (PTCs)

**Figure 13:**
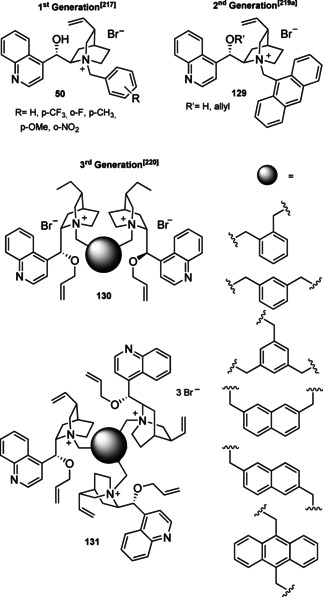
General structures of different generations of Cinchona alkaloid PTCs.

Phase‐transfer catalysts (PTCs) play a key role in asymmetric synthesis, finding from their infancy application in industrial processes. In the light of their importance,^[216]^ especially in terms of scalability and greenness of phase transfer‐run protocols, despite they do not represent an outlier with respect to the adopted classification criteria, PTCs will be treated separately from other organocatalysts. In particular, the first PTC derived from Cinchona alkaloids was applied in 1984 by Dolling and co‐workers from Merck.[^2c^, ^217^] From that moment, the *Cinchona* alkaloid PTCs family has been enlarged and structural modifications have been realized, in order to increase stereocontrol.^[218]^ PTCs derived from Cinchona alkaloids are obtained through formation of a quaternary ammonium cation by reaction of the natural compound with an alkyl or aryl halide. The simplest structures are *N*‐benzyl and *N*‐antracenylmethyl Cinchona PTCs (1^st^ and 2^nd^ generation catalysts; Figure 13). To enlarge substrate scope and risen enantioselectivity, 3^rd^ generation PTC have been developed, attaching two or three molecules of a *Cinchona* alkaloids to an aromatic spacer.

**Scheme 57:**
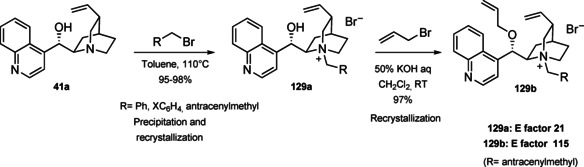
General scheme for the synthesis of 1^st^ and 2^nd^ generation Cinchona alkaloid PTCs.

The synthesis of 1^st^ and 2^nd^ generation catalysts is straightforward and characterized by low *E* factors, meeting, furthermore 1^st^, 2^nd^ and 7^th^ principles of green chemistry (Scheme 57).^[219]^ In fact, the Cinchona alkaloid is directly reacted with benzyl or antracenylmethyl bromide in refluxing toluene and product **129 a** is simply precipitated and recrystallized (*E* factor 21). In the first step, attention should be paid to the aryl bromide since it can cause serious eye, skin and respiratory irritation. Compound **129 a** itself can be used as catalyst or after protection of the hydroxyl group with allyl bromide in 50 % aqueous KOH and CH_2_Cl_2_ at room temperature. Product **129 b** is obtained after crystallization in 97 % yield with a low *E* factor of 115, even though the last synthetic step cannot be considered green because of the employment of a major issue solvent as CH_2_Cl_2_ and of allyl bromide, the latter being very toxic to aquatic life and extremely flammable.

**Scheme 58:**
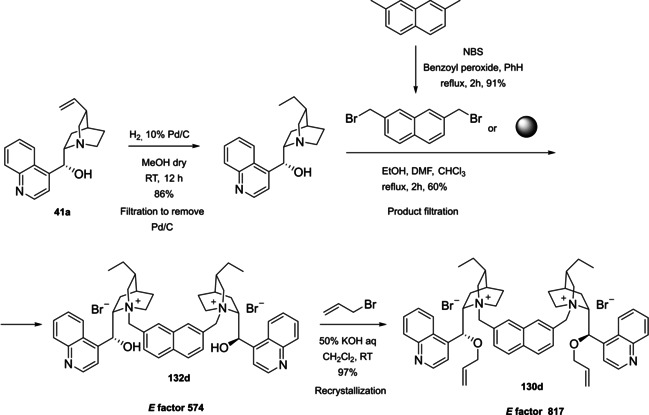
General scheme for the synthesis of 3^rd^ generation Cinchona alkaloid PTCs.

The synthesis of 3^rd^ generation Cinchona PTCs is more demanding from the point of view of the *E* factor. In fact, two additional synthetic steps are necessary to build up the spacer and to dehydrogenate the double bond. The reduction is generally realized with H_2_ on Pd/C introducing an additional purification step in order to remove the metal.^[220]^ Once the alkaloid is reacted with the spacer, catalyst **132 d** is obtained (Scheme 58) with an *E* factor of 574. As described for 1^st^ and 2^nd^ generation catalysts, the hydroxyl group can be protected with allyl bromide, affording catalyst **130 d**, whose *E* factor reaches a value of 817. Apart for palladium filtration, all the synthetic intermediates are purified by filtration and recrystallization. Except for ethanol, all the solvents employed are classified as major issues solvents.

For large‐scale processes solid‐supported PTCs have been developed, exploiting immobilization of the catalyst on a resin.^[221]^

**Figure 14:**
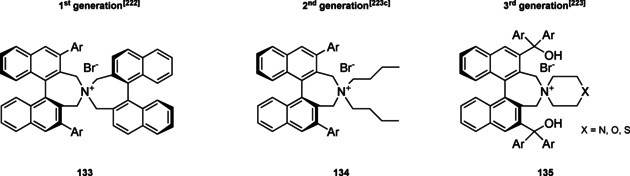
General structures of different generations of Maruoka BINOL‐based PTCs. For 2^nd^ generation catalysts, the structures with n‐butyl groups achieving the best performances.

Another class of PTC derived from BINOL has been presented by Maruoka and co‐workers at the very beginning of the history of modern organocatalysis, for the asymmetric alkylation of amino acid precursors (Figure 14). The synthesis of the catalyst, which possesses a C2‐symmetric and bis‐biaryl structure, is considerably demanding from the point of view of the overall waste amount and the employment of VOCs and unsafe reagents. Later, the structure of the catalyst has been modified by using a combinatorial design approach, despite no significative improvements in terms of the principles of green chemistry were interested by the design of these novel scaffolds.^[222]^ Thus, 1^st^, 2^nd^ and 3^rd^ generation Maruoka catalysts can be distinguished on the basis of the structural complexity of the quaternary ammonium salt. First generation structures, commercially available under the name of Maruoka catalyst®, are tetra‐benzylic *bis*‐biaryl quaternary ammonium salts. Both second and third generation result from combinatorial design and bear just a single biaryl moiety, while the counterpart is constituted by two alkyl groups in the 2^nd^ generation and by a heterocyclic 6‐membered ring in the 3^rd^ generation catalysts, which has also a different substitution pattern in the 3,3’‐positions (Figure 14).^[223]^ Second‐generation ammonium salts are commercially available under the name of Simplified Maruoka catalyst. Third generation salts are commercially available too.

**Scheme 59:**
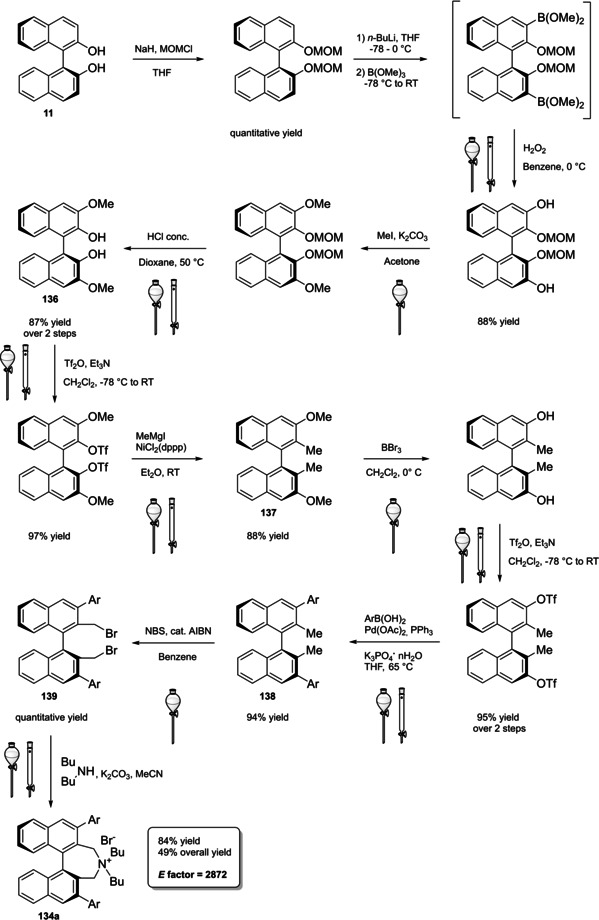
Scheme for the synthesis of Simplified Maruoka catalyst **134 a**.

Since the synthetic routes towards 1^st^ and 2^nd^ generation catalysts–follow a well‐established scheme presenting minimum variability just in the last step, only the synthesis of representative catalyst **134 a** will be analyzed in detail (Scheme 59). Similar to multistep syntheses of several classes of Brønsted acid organocatalysts, the route to compound **134 a** includes cycles of introduction and removal of protecting groups, in particular for the transformation of **11** into compounds **136**–**138**, which means noncompliance to the 1^st^, 2^nd^ and 5^th^ principle. Moreover, numerous major issue solvents are employed throughout the various steps, including benzene. Despite a very good overall yield of 49 %, the recurrence of extractive and chromatographic purifications also gives an important contribution to the high final *E* factor value of 2872. For the synthesis of the corresponding 1^st^ generation catalyst **133 a**, an even higher *E* factor value should be expected, due to the necessity of preparing from **11** a binaphthyl amine to react with dibromide **139**, through a further multistep synthesis.

## 8 Impact of Catalyst Synthesis and Global *E* factor (*E*_g_ Factor)

The 9^th^ principle of green chemistry brings out catalysis as a strategy to fulfill energy efficiency, atom economy and waste reduction, thus directly retrieving the other principles.^[3]^ Consequently, in the light of the *E* factor calculated for the synthesis of the catalyst, one should rethink the question without taking for granted the green advantages of catalysis a priori. With respect to asymmetric organocatalysis, the ideal reaction should be carried out at room temperature, leading to a single stereoisomer, possibly avoiding large amounts of solvents, filtering materials and reactants in excess. Green aspects of the catalyst itself mostly rely on its recyclability rather than on a sustainable synthetic protocol.^[224]^ However, even if excellent product selectivity is obtained at room temperature, the overall process may not be straightforwardly extended on an industrial scale if the catalyst itself cannot be conveniently prepared. Herein, the global *E* factor (*E*_G_ factor) will be introduced as a new green chemistry metric to take into account the synthetic route to the catalyst. Thus, in order to provide an idea of the effective impact that organocatalysts with a very complex structure have on a chemical process, selected examples will be presented and compared according to their *E*_G_ factor.

### 8.1 Green diastereo‐ and enantioselective aldol reaction

**Scheme 60:**
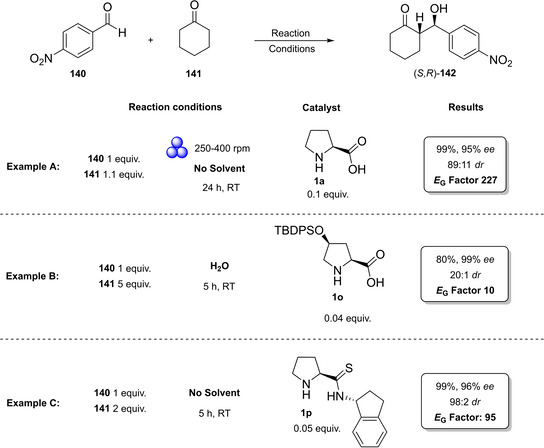
Green diastereo‐ and enantioselective aldol reaction.

The asymmetric aldol reaction between cyclohexanone (**140**) and 4‐nitrobenzaldehyde (**141**) has been chosen as the model reaction, since a plethora of green chemistry methodologies are present in the literature.^[225]^ The catalysts of choice for the organocatalytic version of this reaction are **1 a** or its derivatives, which can be employed in common organic solvents, water,^[226]^ and non‐conventional solvents such as ethyl carbonate^[227]^ and natural deep eutectic solvents (NADES).^[228]^ We will compare the *E*_G_ factors obtained in the conditions depicted in Scheme 60. In example A, the natural amino acid **1 a** is employed under solvent‐free ball‐milling condition;^[229]^ in example B the TBDPS‐protected proline **1 o** is applied in water,^[226]^ while in example C prolinothioamide **1 p** derived from proline is used in solvent‐free condition.^[230]^ In order to be able to compare the three *E* factors, we will not take into account the purification by column chromatography, since *anti‐*aldol product (*S,R*)*‐* **142** can be purified by distillation as demonstrated by Hayashi et al.^[226]^ in their in water conditions. As shown in Scheme 60, the lowest *E*_G_ factor is 10 and it is obtained in the presence of water as the solvent (example B), the preparation of catalyst **1 n** is straightforward, and it has a minimum impact on the entire process. Increasing the complexity of the catalyst it is observed an increase of more than nine times in the *E*_G_ factor (see example C; *E*_G_ factor 95). The preparation of prolinothioamide **1 p** requires, in fact, three consecutive steps. Anyway, the absence of chromatographic purifications and the preference for **1 p** crystallization helps on keeping the *E*_G_ factor below 100. Moreover, organocatalyst **1 p** can be easily recovered from the aldol reaction mixture applying a simple acid/base extraction followed by crystallization. Surprisingly, the higher *E*_G_ factor is obtained under ball‐condition condition (example A, *E*_G_ factor 227), even though the natural amino acid **1 a** is employed as catalyst. A limitation of the ball‐milling is, in fact, the employment of a large amount of solvent in the workup procedure to wash off the aldol product **142** form the zirconia balls employed in the ball‐milling vessel. The last example shows that the employment of natural compounds as catalyst it is not able alone to assure the respect of the green chemistry principles, but all the procedures should be rethought in this point of view.

### 8.2 Diastereo‐ and enantioselective Michael addition

**Scheme 61:**
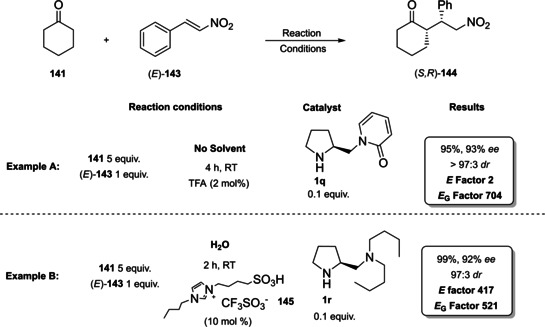
Green diastereo‐ and enantioselective Michael conjugate addition.

In analogy to the case of the aldol reaction, a variety of examples of green organocatalytic asymmetric Michael conjugate addition of cyclohexanone **141** to *(E)‐*nitrostyrene **143** are available in the literature (Scheme 61).^[231]^ Proline derivatives (diamines or prolinamides in most cases) represent the privileged scaffolds to carry out this transformation in excellent yield, stereoselectivity and eco‐friendly fashion. Nevertheless, the synthesis of the organocatalyst scaffold turns out to be importantly influent in the total waste amount and, therefore, in the greenness of the process. As a matter of fact, the synthesis of prolinamide **1 q** presents a high *E* factor of 9263, due to a low overall yield and multiple extractive and chromatographic workups, including, among other, the employment of chlorinated solvents. Thus, despite **1 q** promotes a quick and efficient room temperature Michael reaction between **141** and (*E*)*‐* **143** in solventless conditions (*E* factor 2), the preparation of only 17.8 mg of the catalyst actually gives the major contribution to the total waste, that enhances the value of the E_G_ factor to 704 (Scheme 61).^[232]^ Analogous results in terms of yield and stereoselectivity for (*S*,*R*)*‐* **144** can be observed when 10 mol% of catalyst **1 r** (*E* factor 923) are employed in aqueous media and in the presence of an equimolar amount of the acidic ionic liquid co‐catalyst **145** (*E* factor 144).^[233]^ However, despite synthetic and purification steps shall be priorly considered for both **1 r** ^[234]^ and **145**,^[235]^ they only add a 25 % additional waste, therefore an E_G_ factor of 521 can be calculated in response to an *E* factor of 417. In this case, the relatively high *E* factor value is practically due to the column chromatography used to purify product (*S,R*)*‐* **144**.

### 8.3 Atroposelective synthesis of BINOL‐like non‐C_2_ symmetrical biaryls

**Scheme 62:**
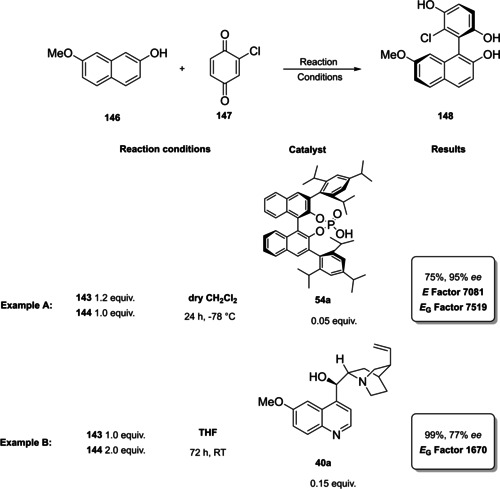
Atroposelective synthesis of BINOL‐like non C2‐symmetrical biaryl **147**.

A good measure of the impact that a complex organocatalyst may have on the overall process can be given by the comparison of two recent atroposelective protocols for the synthesis of non‐C_2_ symmetrical BINOL‐like biaryls by Bella and co‐workers^[5a]^ and Tan and co‐workers.^[236]^ In both cases reactions are performed in not strictly green conditions and purification by column chromatography strongly affects the final *E*_G_ factor values (Scheme 62). The chosen reference biaryl product **148** can be obtained by either chiral base‐ or acid‐mediated addition of 7‐methoxy‐2‐naphthol (**146**) to 2‐chloro‐1,4‐dibenzoquinone (**147**). However, apart from mechanistic details, the catalyst choice is also reflected on the resulting optimal reaction conditions. With **54 a** as the catalyst (example A, Scheme 62), a high degree of enantiopurity (95 % *ee*) of the biaryl adduct is ensured, with a yield limited to 75 %. This detail makes necessary a chromatographic purification, which dramatically impacts the overall waste amount, resulting in an important *E* factor of 7081. Moreover, the reaction is performed in dry CH_2_Cl_2_ at −78 °C, implying major issues related to volatile and chlorinated solvents (5^th^ principle), to the necessity of inert atmosphere (1^st^ and 3^rd^ principle) and low temperatures (6^th^ principle). If one takes into account the synthesis of the BINOL‐based catalyst, an even higher *E*_G_ factor of 7519 is obtained. This means that the 6,2 % additional waste is only due to the synthesis of 5 μmol of the catalyst employed.

Oppositely, the employment of naturally occurring quinine **40 a** (example B), despite in higher loading than **54 a** (15 mol % against 5 mol %), has minor impact on the total waste amount, since no synthesis of the catalyst is required; thus, the *E*_G_ factor coincides with the *E* factor. This procedure is also preferable for its energy efficiency, due to virtually quantitative yield and satisfactory 77 % *ee* obtained at room temperature. The only true weak point is represented by the use of a classical VOC such as THF, despite in this case room for improvement is offered by the possibility of replacing it with biomass‐derived 2‐methyl THF, which has often shown to be a suitable green substitute of THF as well as of other apolar solvents.^[237]^ The relatively high *E* factor of 1670 can be attributed to the use of a pad of silica gel and a moderate volume of eluent employed in the filtration of the crude, which is necessary in order to separate the product from exhausted catalyst and boronate trifluoroacetic salts resulting from the reductive workup of the reaction.

## 9 Conclusions

With reference to the relationship between green chemistry and asymmetric organocatalysis, it may appear that considerable attention has been devoted to developing greener organocatalytic protocols;^[238]^ on the contrary, in this Review it has been shown that catalyst design has rather been oriented towards growing degree of molecular complexity, with poor or even no attention to sustainability aims. The great number of organocatalyst scaffolds developed in academia does not seem to take into account a possible application on an industrial scale, considering the scarce fulfilling of their synthetic routes of the 12 principles of green chemistry. In the light of this perspective, the 9^th^ principle would assume much more significance if green routes to new catalysts were designed, or even if synthetic routes to existing ones were rethought in terms of the other 11 principles. As we show in Chapter 8, the catalyst *E* factor can have a high impact on the overall process. In parallel, despite not reflected by this parameter, catalytic processes and methodologies would certainly benefit from the choice of cheap and renewable starting materials, together with non‐hazardous reactants and mild or simple reaction conditions during the catalyst design phase. In our hopes, the critical aim of this Review may represent a hint to optimize the greenness of multi‐step syntheses of the best performing organocatalysts, as well as to develop new efficient and more sustainable scaffolds. To conclude, in a hopefully not too far future, the introduction of greenness and sustainability as novel cardinal principles of catalyst design would also represent a benign expedient towards a major industrial applicability of asymmetric organocatalysis.

## Conflict of interest

The authors declare no conflict of interest.

## Biographical Information

*Achille Antenucci was born in Campobasso, Italy, in 1991. He obtained both his BS and MS degrees cum laude in chemistry from “Sapienza” University of Rome, Italy, in 2013 and 2016, respectively. In 2019, he completed his PhD at the same institution, working on the development of new green strategies based on Brønsted and Lewis acid catalysis for organic synthesis. In the same year, he joined the group of Prof. Dughera as a postdoctoral research fellow at the University of Turin (Italy), where he is currently working on sustainable synthesis and catalysis*.

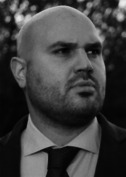

## Biographical Information

*Stefano Dughera is an Associate Professor at the Department of Chemistry University of Turin (Italy). His research lines, always linked to organic synthesis, are focused on sustainable synthesis and catalysis. In particular, he studied several strong organic Brønsted acids as safe and green homogeneous catalysts. Moreover, homogeneous and heterogeneous chiral Brønsted acid catalysts which C2 symmetry were designed. These adducts turned out to be excellent chiral catalysts in multicomponent reactions, usually in green conditions*.

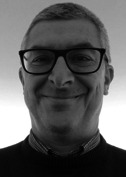

## Biographical Information

*Polyssena Renzi completed her PhD in 2014 at Sapienza University of Rome (Italy) working on the development of novel strategies for asymmetric synthesis. In 2015, she joined the group of Prof. R. M. Gschwind (Regensburg University, Germany) as a postdoctoral fellow, where she dealt with mechanistic investigations of organocatalyzed reactions by means of NMR techniques. Since February 2020, she is Research Associate at the University of Turin, where she works in the field of photocatalysis, in the synthesis of molecules for BNCT applications and metal complexes for the oxygen reduction reaction*.